# Integrative Analysis of the Role of *TP53* in Human Pan-Cancer

**DOI:** 10.3390/cimb45120601

**Published:** 2023-11-29

**Authors:** Tingting Liu, Jin Du, Xiangshu Cheng, Jianshe Wei

**Affiliations:** Institute for Brain Sciences Research, School of Life Sciences, Henan University, Kaifeng 475004, China; ltt0808@henu.edu.cn (T.L.); dujindoctor@outlook.com (J.D.)

**Keywords:** pan-cancer, *TP53*, mutation, immunity, therapy

## Abstract

Tumor protein P53 (*TP53*) is an important tumor suppressor gene in humans. Under normal circumstances, *TP53* can help repair mutated genes, or promote the death of cells with severe gene mutations (specifically, *TP53* prevents cells from arrest in the G1/S phase when deoxyribonucleic acid (DNA) is damaged and promotes apoptosis if not repaired), and prevents normal cells from becoming malignant cells. *TP53* mutations affect its tumor suppressor function, leading to the development of malignant tumors. In this study, using a public database, we explored the pan-cancer expression of *TP53*, its impact on patient survival and prognosis, the types of gene mutations, its correlation with immunity, and its regulation of other transcription factors and micro RNA (miRNA). The docking sites of therapeutic drugs and key amino acid sites of action provide a basis for future targeted therapies. *TP53* has important biological functions in the human body. This study provides a theoretical basis for clinical *TP53* gene therapy.

## 1. Introduction

Tumor protein P53 (*TP53*, also known as *p53*) is a tumor suppressor gene located on the short arm of human chromosome 17. It encodes and expresses *TP53* protein, which is an important cell cycle factor and tumor suppressor [1]. *TP53* is a transcription factor that directly regulates the expression of 500 genes and is associated with important biological functions; apart from its classical function in cell cycle arrest, deoxyribonucleic acid (DNA) repair, apoptosis, and senescence, it also supervises processes such as cellular plasticity, self-renewal, and differentiation [2,3,4]. Under normal/physiological conditions, *TP53* is a tumor suppressor gene that plays a crucial role in maintaining the integrity of the genome. It functions as a transcription factor that regulates the expression of numerous genes involved in cell cycle arrest, DNA repair, apoptosis, and senescence. *TP53* acts as a guardian of the genome by detecting DNA damage and initiating the appropriate response to prevent the propagation of damaged cells [5]. However, the protein expressed by a mutated *TP53* gene has certain structural and functional defects, loses the *TP53* gene’s original biological characteristics of inhibiting tumor cell growth, and promotes the continuous malignant transformation of tumor cells, which leads to the development of malignant tumors. The high expression of *TP53* in tumors is due to the fact that heat shock protein 90 (HSP90) and HSP70 maintain the stability of mutations in *TP53* in cancer by interacting with the DNA-binding domain of mutated *TP53*. The functions of *TP53* in tumors include genetic instability (promoting amplification and chromosomal instability), the regulation of ferroptosis (which has been shown in most studies to promote the occurrence of iron death) and tumor microenvironment, and the acquisition of cancer stem cells (CSCs) phenotypes. The hallmark feature of CSCs is their ability to produce heterogeneous tumor cells, which are critical in the initiation and progression of cancer [6].

Studies have shown that *TP53* is a common mutated gene in various forms of human cancer. *TP53* is related to 50% of human tumors, including liver, breast, gastric, colorectal, esophageal, and lung cancers [7]. Over the years, the role of *TP53* in other cellular processes, such as metabolism, angiogenesis, immune responses, stem cell maintenance, and tumor–stromal cell crosstalk, has emerged. *TP53* has thus earned a position as an “all-rounder” in cancer biology by being involved in the orchestration of the basic events that must be overcome for cancer initiation and progression, summarized as “the hallmarks of cancer” [8]. In addition to mutations, *TP53* activity can be modulated through other mechanisms. Some viruses can inactivate *TP53*, allowing infected cells to evade immune surveillance and promote viral replication. Additionally, various cellular stressors, such as hypoxia, radiation, and chemotherapy, can stabilize *TP53*, leading to the activation of its downstream target genes and inducing cell cycle arrest or apoptosis. Overall, *TP53* plays a critical role in maintaining genomic stability and suppressing tumor formation under normal conditions. Its dysregulation through mutations or other mechanisms contributes to the development and progression of various pathological conditions, particularly cancer [9,10]. The *TP53* gene is divided into wild type (normal gene) and mutant type, and its products are also of wild type and mutant type [11]. Wild-type *TP53* protein acts as a broad-spectrum tumor suppressor by inhibiting the division of cells with DNA damage and chromosomal aberrations and preventing the transmission of aberrations to daughter cells. In contrast, mutations (deletion) in the *TP53* gene are closely related to the occurrence and development of tumors; therefore, *TP53* is known as a gene guardian [12]. Studies have shown that some environments and diets can directly cause mutations in the *TP53* gene, such as exposure to aflatoxin B1 in foods, so it is necessary to avoid eating moldy foods that may contain aflatoxin [13]. Hepatitis B virus (HBV)- and hepatitis C virus (HCV)-related carcinogenesis is initiated in the context of chronic hepatitis, and progresses to HCC in a multistep process lasting for as long as 30 years [14]. During hepatocellular carcinoma (HCC) progression, synergy among several environmental factors (aflatoxin B1, alcohol consumption, cigarette smoking, hepatotoxic chemical agents) as well as host co-factors (elevated serum androgen levels, genetic polymorphisms, DNA repair enzymes) may lead to the progressive accumulation of multiple genomic changes in hepatocytes. Among these, non-synonymous mutations in the *TP53* gene are well-known cancer drivers for HCC development with variable frequencies depending on the underlying etiology [15]. Mutations in *TP53* in non-melanoma skin cancer are associated with ultraviolet (UV) exposure from the sun [16]. Smoking can cause *TP53* mutations, related to the occurrence of lung cancer [17]. Clinically, *TP53* mutations are associated with poor prognosis in some cancers; however, this remains controversial. Studies show that *TP53* mutations are not restricted to glioblastoma multiforme and may be important in the tumorigenesis of lower-grade astrocytomas and that *TP53* mutations in lower-grade astrocytomas are associated with the loss of chromosome 17p [18]. Notably, the World Health Organization (WHO) incorporated the distinction between mutated and wild-type *TP53* in specific brain tumors [19,20], and *TP53* mutational status can have varying implications for different medulloblastoma groups, divided into sonic hedgehog (SHH)-activated and *TP53*-wild type, and SHH-activated and *TP53*-mutant, for example [21]. Moreover, previous studies have shown *TP53* mutations in primary human brain tumors [22]. Therefore, this study explored the pan-cancer expression of *TP53* based on public databases, mutation status, prognostic analysis, diagnostic receiver operating characteristic (ROC), immune infiltration, regulation of the expression of other transcription factors, and further analyses based on *TP53* gene mutations, so as to better understand the function of *TP53* in human cancers and obtain a more accurate prognosis prediction.

## 2. Material and Methods

### 2.1. Identifying Pan-Cancer Expression of TP53 

We compared the expression and methylation of *TP53* in tumor and normal tissues using the TIMER2 (http://timer.comp-genomics.org/ (accessed on 17 May 2023)) [23], gene expression profiling interactive analysis (GEPIA2) (http://gepia2.cancer-pku.cn/#index (accessed on 18 May 2023)) [24], and UALCAN databases (https://ualcan.path.uab.edu/ (accessed on 18 May 2023)) [25]. The parameters in GEPIA2 were set to *p*-value < 0.05, |log_2_ (fold change)| > 1, and the GEPIA2 database was used to analyze the pathological stage. Z-values represent standard deviations from the median across samples for each cancer type. Log2 spectral count ratio values from the Clinical Proteomic Tumor Analysis Consortium (CPTAC) were first normalized within each sample profile and then across samples.

### 2.2. Survival Prognosis, Mutation, and mRNA Expression Analysis

Across different tumor types, the prognostic values of *TP53*, including overall survival (OS) and disease-free survival (RFS), were determined using the GEPIA2 database, TCGA, and Kaplan–Meier plotter. Heatmap data and survival plots for *TP53* are shown. In addition, we used the cBioPortal tool (https://www.cbioportal.org/ (accessed on 19 May 2023)) [26] and GSCALite database (http://bioinfo.life.hust.edu.cn/web/GSCALite/ (accessed on 19 May 2023)) [27] to explore the mutation frequency, mutation type, site information, and mRNA expression of *TP53* across different tumors. We also assessed the survival values of *TP53* genetic alterations, including OS and RFS, across the different cancers.

### 2.3. Pan-Cancer Clinical Value of TP53 

We downloaded the standardized pan-cancer dataset from the UCSC (https://xenabrowser.net/ (accessed on 20 May 2023)) database: The Cancer Genome Atlas (TCGA) Pan-Cancer (PANCAN, N = 10,535, G = 60,499). Further, we extracted ENSG00000141510 (*TP53*) gene expression data for each sample, and expression values for each of the log2 (x + 0.001) transforms. We built a Cox proportional hazards regression model using the coxph function of the R software package survival (version 3.2-7), and this was used to analyze the relationship between gene expression and prognosis for each tumor. The logrank test was used for statistical testing to obtain prognostic significance. To screen the pan-cancer expression of the *TP53* gene, we visually displayed the results of ROC curve analysis, and AUCs were calculated using the pROC package in R software (version 4.2.1) to determine the predicted values of the *TP53* gene.

### 2.4. Analysis of Immune Infiltration

We analyzed the relationship between *TP53* expression and immune infiltrates across all tumors using the TIMER2 tool. We selected B cells, CD8+ T cells, CD4+ T cells, macrophages, neutrophils, dendritic cells (DC), natural killer cells (NK cells), monocytes, cancer-associated fibroblasts (CAF), endothelial cells, eosinophils, and regulatory T cells (Tregs) for detailed analysis. Seven algorithms, namely TIMER, EPIC, MCPCOUNTER, CIBERSORT, CIBERSORT-ABS, QUANTISEQ, and XCELL, were applied in the analysis of immune infiltration. The gene expression profile of each tumor was extracted and mapped to GeneSymbol, and the immune score of each patient with each tumor was calculated according to gene expression using the R software package ESTIMATE (version 1.0.13, https://bioinformatics.mdanderson.org/public-software/estimate/ (accessed on 21 May 2023)). differences in the significance of analysis were determined using the R software package psych (version 2.1.6), and the Pearson’s correlation coefficients of genes as well as immune invasion scores of individual tumors were calculated to determine the significance of correlation with immune invasion scores. Using the Timer method of the R software package IOBR (version 0.99.9), we reassessed the immune cell invasion score for each patient with each tumor and plotted a survival curve.

### 2.5. Tumor Stemness of TP53

The DNA tumor dryness score was calculated from the methylation characteristics for each tumor; we integrated the sample dryness index and gene expression data and further transformed each expression value with log_2_ (x + 0.001). Finally, we eliminated cancers with less than three samples each, and finally obtained the expression data of 37 cancers. We also analyzed two types of immune checkpoints in each sample, including 26 inhibition sites and 36 activation sites.

### 2.6. Analysis of Single-Cell Sequencing Data

At the single-cell level, we explored the correlation between *TP53* expression and tumor functional status by searching CancerSEA (http://biocc.hrbmu.edu.cn/CancerSEA/ (accessed on 22 May 2023)) [28]. A heatmap was drawn to indicate significant correlations. The top four significantly different functional states (*p* < 0.05) and the T-SNE diagrams of tumors were obtained based on the CancerSEA database.

### 2.7. Enrichment Analysis of TP53-Related Genes

STRING (https://cn.string-db.org/ (accessed on 23 May 2023)) [29] was used to perform the molecular interaction network analysis. Furthermore, GEPIA2 was used to explore Spearman correlations between *TP53* and the selected genes. The miRNA network was selected for analysis using the GSCALite database. The Oncomir (http://www.oncomir.org/ (accessed on 23 May 2023)) database [30] was used to perform the cluster analysis of related miRNAs and pan-cancer survival analysis. At the same time, the wikipathways database (https://www.wikipathways.org/ (accessed on 24 May 2023)) [31] was used to analyze *TP53*-related signaling pathways. The TRRUST database (https://www.grnpedia.org/trrust/Network_search_form.php (accessed on 25 May 2023)) [32] was used to screen *TP53*-related transcription factors. Vakser Lab (http://gramm.compbio.ku.edu/ (accessed on 25 May 2023)) [33] was used to perform docking analysis of *TP53* and its related transcription factors.

### 2.8. Drug Screening and Molecular Docking

The Connectivity Map database (CMap, https://clue.io/query (accessed on 26 May 2023)) [34] was used to screen compounds for *TP53*-related targets, and the DoGSite database (https://proteins.plus/ (accessed on 27 May 2023)) [35] was used to perform molecular docking analysis. The best binding site was found.

### 2.9. Statistical Analysis

In TIMER2, statistical significance calculated using the Wilcoxon test was annotated with a specific number of stars. In GEPIA2, we used the analysis of variance (ANOVA) method to compare tumors with normal samples. Spearman rank correlation coefficients were used to evaluate the correlations between two groups. We used the Kaplan–Meier method to evaluate the relationship between patient prognosis and *TP53* expression or mutation level. *p* < 0.05 was considered a statistically significant difference.

## 3. Results

### 3.1. Aberrant Pan-Cancer Expression of TP53 

**Figure 1:**
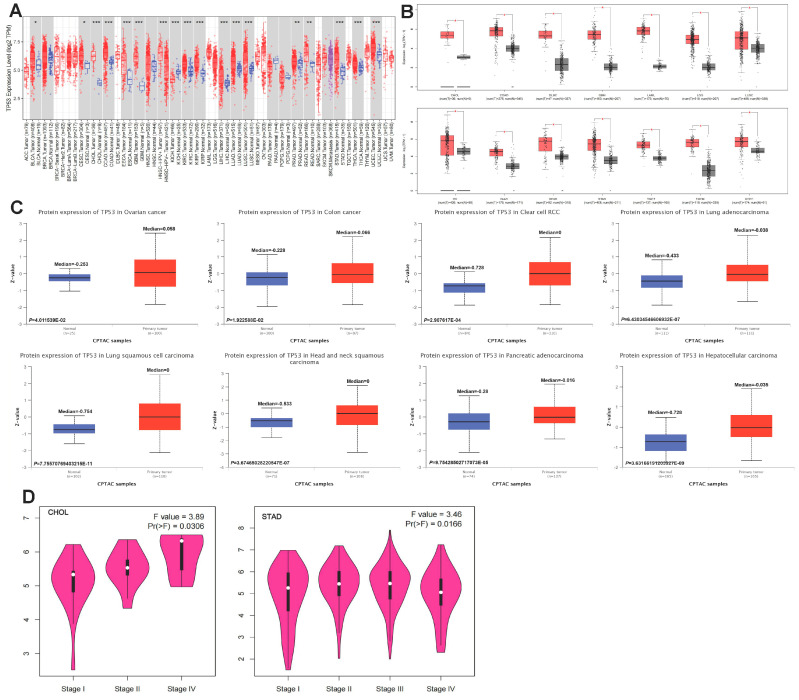
Aberrant pan-cancer expression of *TP53*. (**A**) mRNA level of *TP53* based on the TIMER2 database: blue represents the healthy control group, red represents tumor patients. (**B**) Box plot of *TP53* mRNA level in CHOL, COAD, DLBC, GBM, LAML, LCC, LUSC, OV, PAAD, READ, STAD, TGCT, THYM, and UCEC based on the GEPIA2 database: gray represents the healthy control group, red represents tumor patients. (**C**) The expression of *TP53* in normal tissue and OV, COAD, KIRC, LUAD, LUSC, HNSC, PAAD, and LIHC based on CPTAC: blue represents the healthy control group, red represents tumor patients. (**D**) The expression of *TP53* in each tumor pathological stage. Relationship between *TP53* expression and tumor pathological stage based on GEPIA2. Compared with the healthy control group, * *p* < 0.05; ** *p* < 0.01; *** *p* < 0.001.

In this study, TIMER2 was used to investigate the differential expression of *TP53* by comparing tumors and normal tissues. As shown in Figure 1A, *TP53* expression was significantly upregulated in 15 tumor tissues, namely BLCA (tissue type: bladder urothelial), CESC (cervix), CHOL (bile duct), COAD (colon), ESCA (esophageal), GBM (brain), KIRC (kidney), KIRP (kidney), LIHC (liver), LUAD (lung), LUSC (lung), PRAD (prostate), READ (rectum), STAD (stomach), THCA (thyroid), and UCEC (endometrial). In contrast, *TP53* expression was significantly downregulated in KICH. We further assessed the differential expression of *TP53* in tumor and normal tissues by matching TCGA and GTEx data for several cancers. We found upregulated expression of *TP53* in CHOL, COAD, DLBC, GBM, LAML, LCC, LUSC, OV, PAAD, READ, STAD, TGCT, THYM, and UCEC (Figure 1B, Supplementary Table S1). For the other tumors, there were no significant differences in the expression of *TP53* (Supplementary Figure S1A). To better understand the differential expression, the CPTAC dataset was used to assess *TP53* protein levels from a pan-cancer perspective. As shown in Figure 1C, *TP53* expression was significantly increased in OV, COAD, KIRC, LUAD, LUSC, HNSC, PAAD, and LIHC. For the other tumors, there were no significant differences in the expression of *TP53* (Supplementary Figure S1B). GEPIA2 and TCGA were also used to analyze the relationship between *TP53* expression and tumor pathological stage. As shown in Figure 1D, stage-specific changes in *TP53* expression were observed in two tumor types: CHOL and STAD. In other cancers, there was no clear association between *TP53* expression and pathological stage (Supplementary Figure S1C).

### 3.2. Survival Analysis of TP53 Expression in Pan Cancer

**Figure 2:**
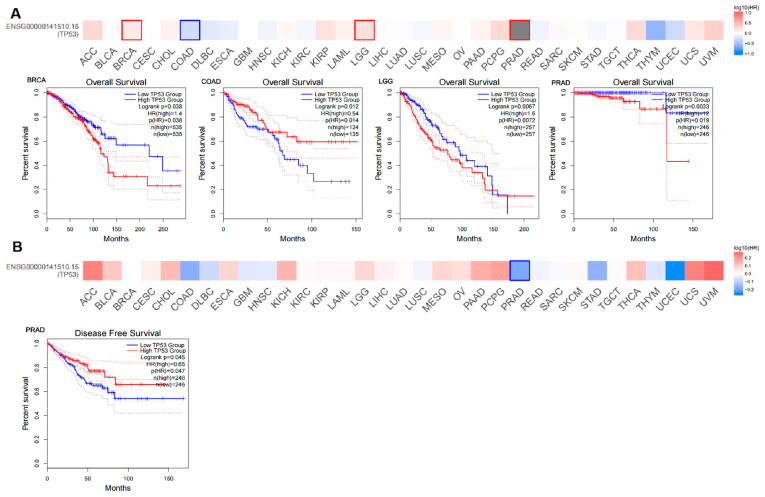
Survival analysis of *TP53* expression in pan cancer (**A**,**B**) The role of *TP53* expression in patient prognosis based on GEPIA2, including OS (**A**) and RFS (**B**). High cutoff (50%) and low cutoff (50%) values were used as the expression thresholds for splitting the high-expression and low-expression cohorts. The cutoff value was as follows: *p*-value < 0.01 and |log_2_ (fold change)| ˃ 1.

We used GEPIA2 to explore the role of *TP53* in patient prognosis, including OS and RFS. In OS, high expression of *TP53* was associated with poor prognosis in patients with BRCA (*p* = 0.038), LGG (*p* = 0.0072), and PRAD (*p* = 0.019). Inversely, high expression of *TP53* was associated with good prognosis in patients with COAD (*p* = 0.014) (Figure 2A). In RFS, high expression of *TP53* was associated with good prognosis in patients with PRAD (*p* = 0.047) (Figure 2B). Furthermore, the Kaplan–Meier plotter tool was used to identify the survival value of *TP53*. As shown in Supplementary Figure S2A, we found that a high *TP53* RNA expression level was associated with poor prognosis in patients with BRCA and COAD. These results indicate the promising role of *TP53* in the prognosis of patients with BRCA and COAD.

### 3.3. Clinical Significance and Prognostic Analysis of TP53

**Figure 3:**
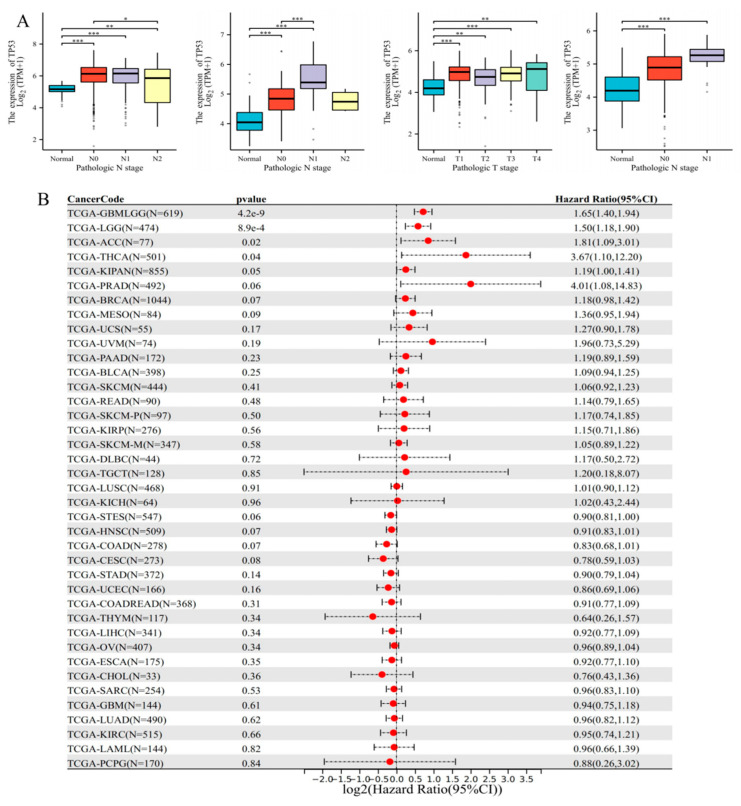
Clinical significance and prognostic analysis of *TP53*. (**A**) Clinicopathologic staging, including N stage in COAD and KIRP, and T and N stages in KIRC. * *p* ˂ 0.05, ** *p* ˂ 0.01, *** *p* ˂ 0.001. (**B**) Pan-cancer prognostic analysis of *TP53* using univariate Cox regression.

*TP53* is highly expressed in most tumors. We analyzed the clinical significance of *TP53* in tumors by clinicopathological T, N, and M stages, and found that there were differences in N stage in COAD and KIRP, and differences in T and N stages in KIRC (Table 1, Figure 3A). Next, we analyzed the prognosis and diagnosis of *TP53* in pan-cancer. The results showed that *TP53* expression in five types of tumors was increased with poor prognosis: TCGA–GBMLGG (N = 619, *p* = 4.2 × 10^−9^, HR = 1.65 (1.40, 1.94)), TCGA LGG (N = 474, *p* = 8.9 × 10^−4^, HR = 1.50 (1.18, 1.90)), TCGA KIPAN (N = 855, HR = 1.19, *p* = 0.05 (1.00, 1.41)), TCGA–THCA (N = 501, *p* = 0.04, HR = 3.67 (1.10, 12.20)), and TCGA–ACC (N = 77, *p* = 0.02, HR = 1.81 (1.09, 3.01)) (Figure 3B, Supplementary Table S3). The diagnostic analysis included ROC curve and logistics analyses. The T, N, and M clinicopathological staging and logistics model of *TP53* are shown in Table 2.

### 3.4. Diagnostic ROC Analysis of TP53

**Figure 4:**
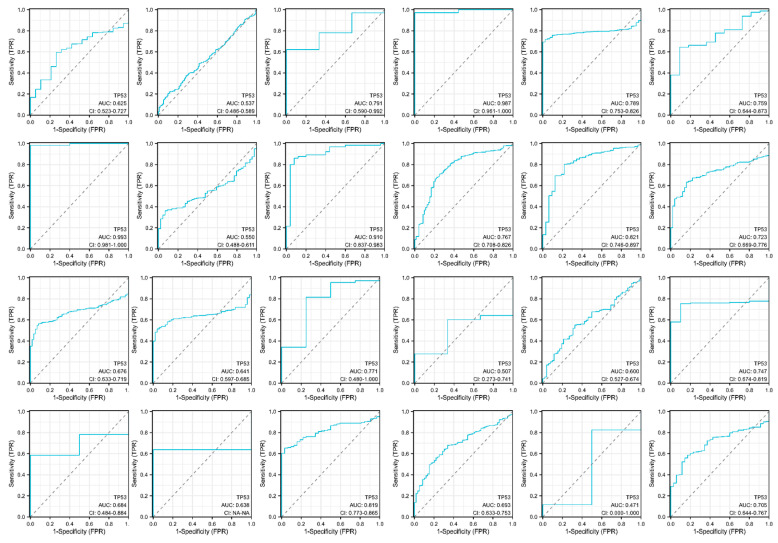
Pan-cancer diagnostic value of the *TP53* gene, including BLCA, BRCA, CESC, CHOL, COAD, ESCA, GBM, HNSC, KICH, KIRC, KIRP, LIHC, LUAD, LUSC, PAAD, PCPG, PRAD, READ, SARC, SKCM, STAD, THCA, THYM, and UCEC.

The value of the *TP53* gene in pan-cancer diagnosis was evaluated using ROC curves. Figure 4 shows the pan-cancer diagnostic values of the *TP53* gene. The ROC analysis showed that the respective areas under the curves (AUCs) for BLCA, BRCA, CESC, CHOL, COAD, ESCA, GBM, HNSC, KICH, KIRC, KIRP, LIHC, LUAD, LUSC, PAAD, PCPG, PRAD, READ, SARC, SKCM, STAD, THCA, THYM, and UCEC were 0.625, 0.537, 0.791, 0.987, 0.789, 0.759, 0.993, 0.550, 0.910, 0.767, 0.821, 0.723, 0.676, 0.641, 0.771, 0.507, 0.600, 0.747, 0.684, 0.638, 0.819, 0.693, 0.471, and 0.705, respectively. The results showed that AUC > 0.5 (except THYM), indicating that the *TP53* gene has good diagnostic value throughout the analyzed cancers.

### 3.5. Pan-Cancer Genetic Alterations of TP53 

**Figure 5:**
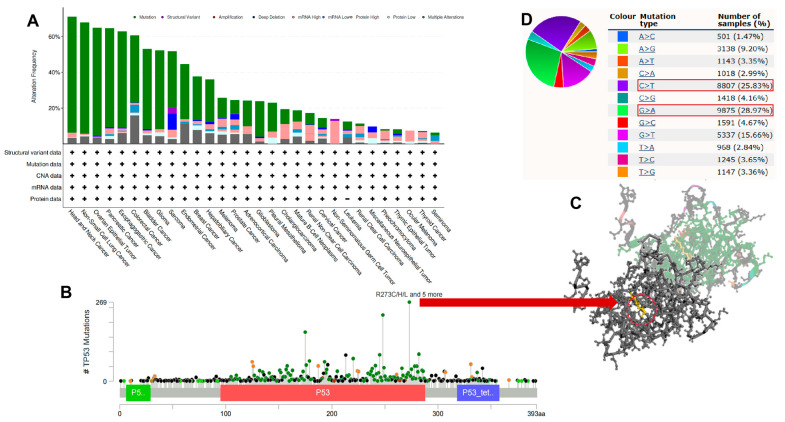
Pan-cancer genetic alterations of *TP53*. (**A**) Pan-cancer mutation status of *TP53* was performed using the cBioPortal tool. (**B**) Main mutation types of *TP53*. (**C**) The R273C/R273H/R273L/R273S/R273P/R273G/R273Lfs*72N274_G279del mutation site was visualized in the 3D structure of *TP53* protein. (**D**) *TP53* base mutation frequency.

According to our analysis, the frequency of *TP53* alterations (64.82%) was highest in undifferentiated HNSC with “mutation” as the primary type. COAD had the highest incidence of the “multiple alterations” type, with a frequency of 15.99%. Non-seminomatous germ cell tumors had the highest incidence of the “mRNA high” type, with a frequency of 9.3%. SARC had the highest incidence of the “structural variant” and “deep deletion” types, with frequencies of 3.53% and 9.02%, respectively (Figure 5A). Patient *TP53* alteration information is shown in Supplementary Table S2. As shown in Figure 5B, there were 4250 mutations in the full sequence of *TP53*. Also, “mutation” seemed to be the main type of genetic alteration and was mainly located within the P53 DNA-binding domain (95-288). For instance, a missense mutation with potential clinical significance, the R273C/R273H/R273L/R273S/R273P/R273G/R273Lfs*72N274_G279del alteration, was detected in many cases (269 mutations), including in LAML, UCS, ACC, LGG, GBM, LUSC, BLCA, LIHC, PRAD, UCEC, LUAD, ESCA, etc. In addition, the AAChange site was visualized in the 3D structure of *TP53* protein (Figure 5C). Most substitution mutations were G to A transitions, followed by C to T transitions (Figure 5D). As shown in Supplementary Figure S2B, genetic alterations in *TP53* showed correlations with poor prognosis (including OS and RFS) in LGG and PRAD. The Chi-square test was used to evaluate the differences in the frequencies of gene mutations in each group of samples, and the results of the mutation landscape are shown in Supplementary Figure S3.

### 3.6. Pan-Cancer mRNA Expression of TP53 

**Figure 6:**
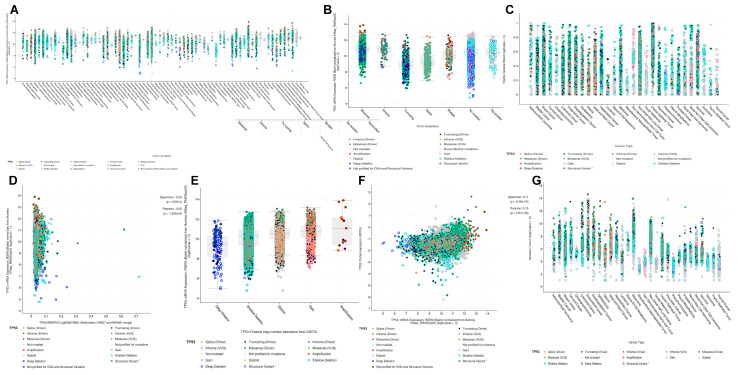
Pan-cancer mRNA expression of *TP53*. (**A**) Pan-cancer mutations of *TP53* mRNA expression, including Splice (Driver), Truncating (Driver), Inframe (Driver), Inframe (VUS), Missense (Driver), Missense (VUS), Not mutated, Not profiled for mutations, Amplification, Gain, Diploid, Shallow Deletion, Deep Deletion, Structural Variant, Not profiled for CNA, and Structural Variants. (**B**) mRNA expression under different mutations of *TP53*: the horizontal coordinate represents *TP53* mutation type, including Missense, Inframe, Truncating, Splice, Multiple, No mutation, and Not profiled, and the vertical coordinate represents mRNA expression. (**C**) Pan-cancer alterations and mutations in *TP53* fragments: the horizontal coordinate represents the various tumors and the vertical coordinate represents the *TP53* alterations and types of alterations. (**D**) *TP53* methylation affects mRNA expression: the horizontal coordinate represents *TP53* methylation [WRAP53 (cg06587969): methylation (HM27 and HM450 merge)] and the vertical coordinate represents mRNA expression. (**E**) Relationship between mRNA and putative copy number of *TP53*: the horizontal coordinate represents the *TP53* copy number, including Deep Deletion, Shallow Deletion, Diploid, Gain, and Amplification, and the vertical coordinate represents mRNA expression. (**F**) Relationship between *TP53* mRNA expression and protein expression: the horizontal coordinate represents *TP53* mRNA expression and the vertical coordinate represents protein expression. (**G**) Pan-cancer *TP53* mutation counts: the horizontal coordinate represents the various tumors and the vertical coordinate represents the count of *TP53* mutations.

Figure 6A shows the pan-cancer expression of *TP53* mRNA, mutation sites, and copy number alterations (CNAs). *TP53* mRNA was most highly expressed in OV, UCEC, LUSC, DLBC, ESCA, and COAD. The *TP53* mutations included Y163C, P278T, Y220C, H193P, T253N, T253A, and I195N. The most common CNA mutations included amplification, gain, diploid, and shallow deletions. As shown in Figure 6B depicting the expression of *TP53* mRNA under different mutation situations, the most common type of mutation in *TP53* corresponding to the highest mRNA value was missense (VUS), and the CNA mutation was amplified. As shown in Figure 6C, pan-cancer alterations in the *TP53* gene fragment were found, with greater alterations in ACC, OV, BRCA, UVM, and COAD. The mutation sites were R196*, S149Ffs*32, Q38Kfs*6, and E285*. The CNA types were diploid and shallow deletion. Figure 6D shows *TP53* methylation (WRAP53 (cg06587969): methylation (HM27 and HM450 merge)), mRNA expression, mutation location, and CNAs. The highest mRNA expression value for *TP53* methylation was log10 = 0.02201, corresponding to the mutation point Y163C. The CNA referred to amplification. R273H was the mutation point of *TP53*, with a high log10 = 0.620327 expression value, and the CNA was shallow deletion. As shown in Figure 6E, regarding the relationship between the putative CNA and mRNA expression of *TP53*, the mutation site with the highest mRNA expression was Y163C, and both putative CNA and CNA were amplified. As shown in Figure 6F, the relationship between mRNA expression and *TP53* protein showed that the highest mRNA expression value was negatively correlated with protein expression when the mutation site was Y163C and the CNA was amplified. The highest protein expression value was positively correlated with mRNA expression when the mutation site was R273S and the CNA was diploid. As shown in Figure 6G depicting the number of pan-cancer *TP53* mutations, mutation sites, and CNAs, the highest numbers of *TP53* mutations were seen in UCEC, UVM, and GBM. The mutation sites were H168Cfs*8, R273C, X125_splice, E56*, X25_splice, and Y234C. The CNAs included gain, diploid, and shallow deletion. The pan-cancer mRNA expression, mutation sites, methylation, and protein expression of *TP53* are shown in Supplementary Table S4.

### 3.7. Pan-Cancer DNA Methylation of TP53 

**Figure 7:**
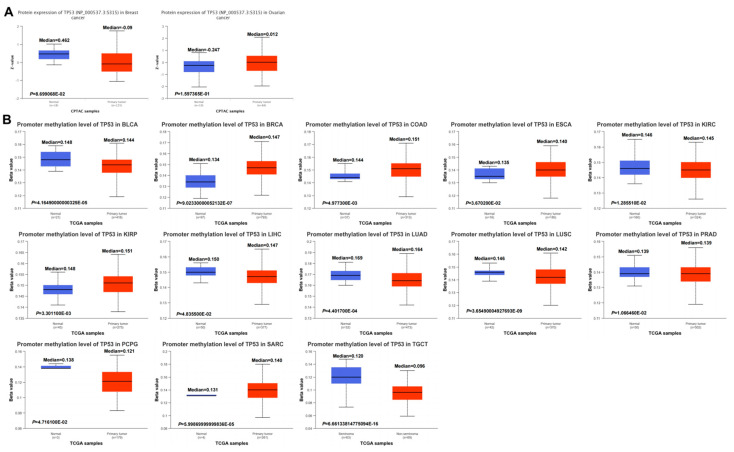
Pan-cancer protein phosphorylation and DNA methylation of *TP53*. (**A**) CPTAC indicated the phosphorylation levels of PDHA1 at S315. (**B**) DNA methylation of *TP53* between normal and primary tumor tissues based on the UALCAN database: blue represents the healthy control group, red represents tumor patients.

We further explored the phosphorylation of *TP53* in normal and primary tumor tissues. However, using the CPTAC dataset, we found no pan-cancer differences in *TP53* phosphorylation (Figure 7A). In multiple cancers, DNA methylation throughout the genome is an epigenetic modification that contributes to the regulation of cancer-associated genes. However, the underlying role of *TP53* methylation in various cancers remains unclear. In our study, we demonstrated decreased promoter methylation levels of *TP53* in BLCA, KIRC, LIHC, LUAD, LUSC, PRAD, PCPG, SARC, and TGCT and increased promoter methylation levels of *TP53* in BRCA, COAD, ESCA, and KIRP (Figure 7B). No obvious changes in the methylation values of *TP53* were observed in other cancers (Supplementary Figure S4).

### 3.8. Pan-Cancer Roles of TP53 in Immune Infiltration 

**Figure 8:**
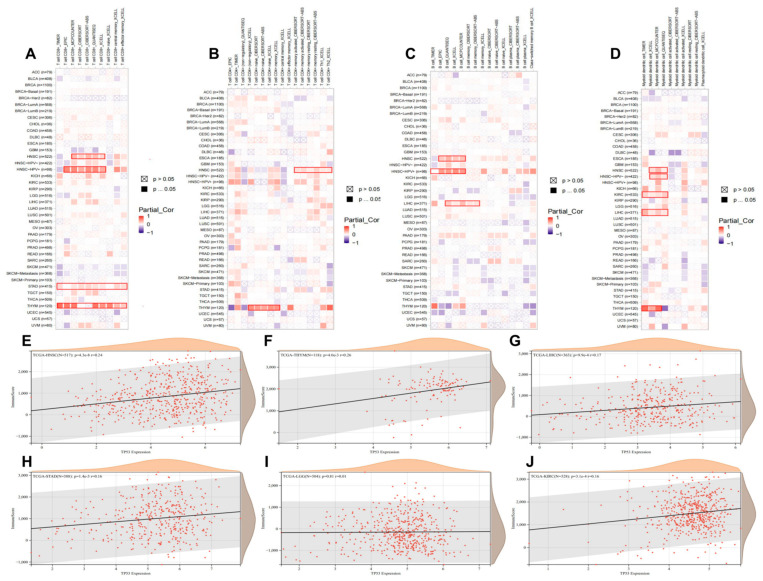
Roles of *TP53* in immune infiltration and immune scores in all TCGA tumor types. (**A**–**D**) Correlation heatmap between *TP53* expression and tumor infiltrating immune cells across different cancer types, including CD8+ T cells (**A**), CD4+ T cells (**B**), B cells (**C**), and myeloid dendritic cells (**D**). A positive correlation is marked as red, while a negative correlation is marked as blue. Non-significant correlations values are marked with a cross. (**E**–**J**) The correlation between *TP53* expression and immune infiltration in HNSC (R = 0.24) (**E**), LIHC (R = 0.26) (**F**), THYM (R = 0.17) (**G**), STAD (R = 0.16) (**H**), LGG (R = 0.01) (**I**), and KIRC (R = 0.16) (**J**): the horizontal coordinate represents *TP53* expression and the vertical coordinate represents immune score.

Here, we explored the potential correlation between *TP53* expression and tumor-infiltrating immune cells by performing a comprehensive analysis. Seven algorithms, namely TIMER, EPIC, MCPCOUNTER, CIBERSORT, CIBERSORT-ABS, QUANTISEQ, and XCELL, were applied to estimate immune infiltration in different tumor types. As shown in Figure 8A–D, there were positive correlations between *TP53* expression and CD8+ T cells, CD4+ T cells, B cells, and myeloid dendritic cells (DC) in HNSC, LIHC, THYM, STAD, LGG, and KIRC. Supplementary Figure S5A shows that pan-cancer *TP53* expression was negatively correlated with the immune cells. At the same time, we also investigated the correlation between *TP53* expression and immune infiltration in HNSC (R = 0.24), LIHC (R = 0.26), THYM (R = 0.17), STAD (R = 0.16), LGG (R = 0.01), and KIRC (R = 0.16). The results showed that *TP53* expression was positively correlated with immune cell infiltration (Figure 8E–J). Information on *TP53* expression and tumor-related immune infiltration is shown in Supplementary Table S5.

### 3.9. Analysis of Tumor Stemness and Immune Checkpoints

The tumor suppressor *TP53* maintains an equilibrium between self-renewal and differentiation to sustain a limited repertoire of stem cells for the proper development and maintenance of tissue homeostasis. The inactivation of *TP53* disrupts this balance and promotes pluripotency and somatic cell reprogramming. A few reports in recent years have indicated that prevalent *TP53* oncogenic gain-of-function (GOF) mutations further boost the stemness properties of cancer cells [36]. Stem cells are a rare population of cells that can perpetuate themselves through self-renewal and can produce mature cells of a tissue by differentiation [37]. We observed a significant association with *TP53* in 14 tumors, with significant positive correlations in 3 tumors (LGG (N = 507) (R = 0.186, *p* = 2.54 × 10^−5^), LAML (N = 167) (R = 0.284, *p* = 1.98 × 10^−4^), THYM (N = 119) (R = 0.415, *p* = 2.65 × 10^−6^)) and significant negative correlations in 11 tumors (BRCA (N = 1080) (R = −0.099, *p* = 0.001), SARC (N = 253) (R = −0.289, *p* = 3.19 × 10^−6^), KIRP (N = 283) (R = −0.147, *p* = 0.013), KIPAN (N = 860) (R = −0.398, *p* = 4.52 × 10^−34^), KIRC (N = 512) (R = −0.372, *p* = 3.01 × 10^−18^), LIHC (N = 366) (R = −0.237, *p* = 4.31 × 10^−6^), THCA (N = 499) (R = −0.263, *p* = 2.44 × 10^−9^), TGCT (N = 147) (R = −0.223, *p* = 0.006), PCPG (N = 176) (R = −0.239, *p* = 0.001), SKCM (N = 102) (R = −0.355, *p* = 2.47 × 10^−4^), KICH (N = 65) (R = −0.257, *p* = 0.039)) (Supplementary Figure S5B). The pan-cancer relationship between *TP53* and immune checkpoints included 24 inhibition sites and 36 activation sites. The inhibition sites mainly included CD276, VEGFB, IL10, LAG3, and PDCD1, whereas the activation sites included HMGB1, TNFSF4, BTN3A1, TNF, ICAM1, and CD27 (Supplementary Figure S5C).

### 3.10. Expression Pattern of TP53 in a Single Cell and Its Relationship with Cancer Functional Status

**Figure 9:**
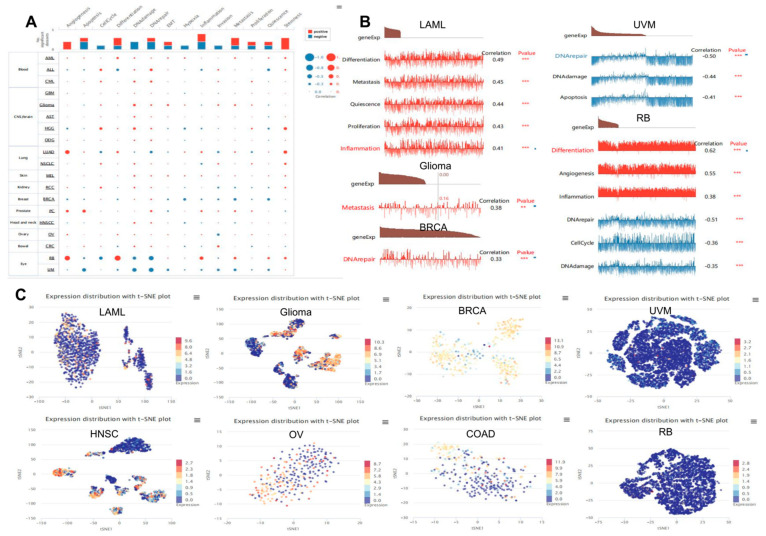
Expression pattern of *TP53* at the single-cell level and its relationship with cancer functional status. (**A**) Correlation between *TP53* expression and different tumor functional status is displayed as a heatmap based on the CancerSEA database. (**B**) Correlation between *TP53* expression and different functional states. In different tumors, red represents a positive correlation between *TP53* and functional status, while blue represents a negative correlation with function status. (**C**) *TP53* expression profiles at single-cell levels in LAML, Glioma, BRCA, UVM, HNSC, OV, COAD, and RB by T-SNE diagrams. ** *p* < 0.01; *** *p* < 0.001.

Single-cell sequencing technology has been used to study the internal heterogeneity of tumor cells, tumor invasion and metastasis, tumor treatment and drug resistance, and evolution of the tumor microenvironment [38,39]. We searched the CancerSEA website to verify the expression of *TP53* at the single-cell level in different cancers and its relationship with the tumor’s functional status. In Figure 9A and Supplementary Table S6, the heatmap shows that *TP53* was strongly correlated with tumor functional status in most cancer types. Figure 9B and Supplementary Table S7 show the relationship between *TP53* expression and LAML, Glioma, BRCA, UVM, and RB. *TP53* expression profiles are shown at single-cell levels in LAML, Glioma, BRCA, UVM, HNSC, OV, COAD, and RB using T-SNE diagrams (Figure 9C). These results suggest that *TP53* may play a crucial role in the biological processes of cancer progression, including showing positive correlations with differentiation, metastasis, inflammation, proliferation, and quiescence, and negative correlations with DNA repair, DNA damage, cell cycle, and apoptosis.

### 3.11. Co-Expression Network of TP53

**Figure 10:**
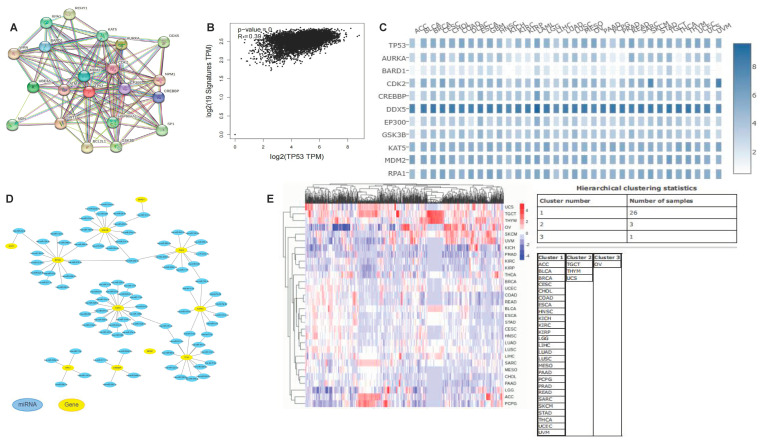
Co-expression network and miRNA analysis of *TP53*. (**A**) Co-expression network of SLC31A1. (**B**) SLC31A1-correlated genes based on GEPIA2. (**C**) Heatmap representation of the expression correlation between *TP53* and ten *TP53*-correlated genes, AURKA, BARD1, CDK2, CREBBP, DDX5, EP300, GSK3B, KAT5, MDM2, and RPA1, in different tumors. (**D**) The miRNA expression of genes. (**E**) Cluster analysis of hsa-let-7e-5p, hsa-miR-98-5p, hsa-miR-221-3p, hsa-miR-222-3p, hsa-miR-380-5p, and hsa-miR-485-5p.

The *TP53*-related proteins were aurora kinase A (AURKA), BRCA-associated RING domain 1 (BARD1), cyclin-dependent kinase 2 (CDK2), CAMP responsive element binding protein (CREBBP), DEAD-box helicase 5 (DDX5), E1A-binding protein P300 (EP300), glycogen synthase kinase 3β (GSK3β), lysine acetyltransferase 5 (KAT5), MDM2 proto-oncogene (MDM2), and replication protein A1 (RPA1). The PPI network contained 21 nodes and 120 edges, with an average node degree of 11.4 and the average local clustering coefficient was 0.738 (Figure 10A). As shown in Figure 10B and Supplementary Figure S6A, *TP53* showed good correlations with the interacting proteins. The heatmap demonstrated that *TP53* had strong positive correlations with the ten aforementioned genes in most cancer types (Figure 10C). As shown in Figure 10D, the miRNAs that were strongly associated with *TP53* included hsa-let-7e-5p, hsa-miR-98-5p, hsa-miR-221-3p, hsa-miR-222-3p, hsa-miR-380-5p, and hsa-miR-485-5p. Cluster analysis was performed on the above miRNAs, which were divided into three categories; a positive correlation is marked in red, while a negative correlation is marked in blue (Figure 10E). Supplementary Figure S6B shows the results of pan-cancer survival curve analysis for these miRNAs. High expression of hsa-let-7e-5p and hsa-miR-98-5p was associated with a long survival time, while high expression of hsa-miR-221-3p, hsa-miR-222-3p, hsa-miR-380-5p, and hsa-miR-485-5p was associated with a shorter survival time. At the same time, some studies have shown that these miRNAs are related to pan-cancer cell proliferation, migration, and immune infiltration [40,41,42,43]. These results indicate that miRNAs play an important role in the regulation of pan-cancer prognosis. Thus, they have the potential to serve as pan-cancer biomarkers. The signaling pathways involved included the pentose phosphate, glycolysis, tricarboxylic acid cycle, T cell activation, ferroptosis, DNA response damage, Wnt signaling, mitogen-activated protein kinase 1 (MAPK) signaling, transforming growth factor (TGF), and cell cycle pathways, among others (Supplementary Figure S7).

### 3.12. TP53-Related Transcription Factors

**Figure 11:**
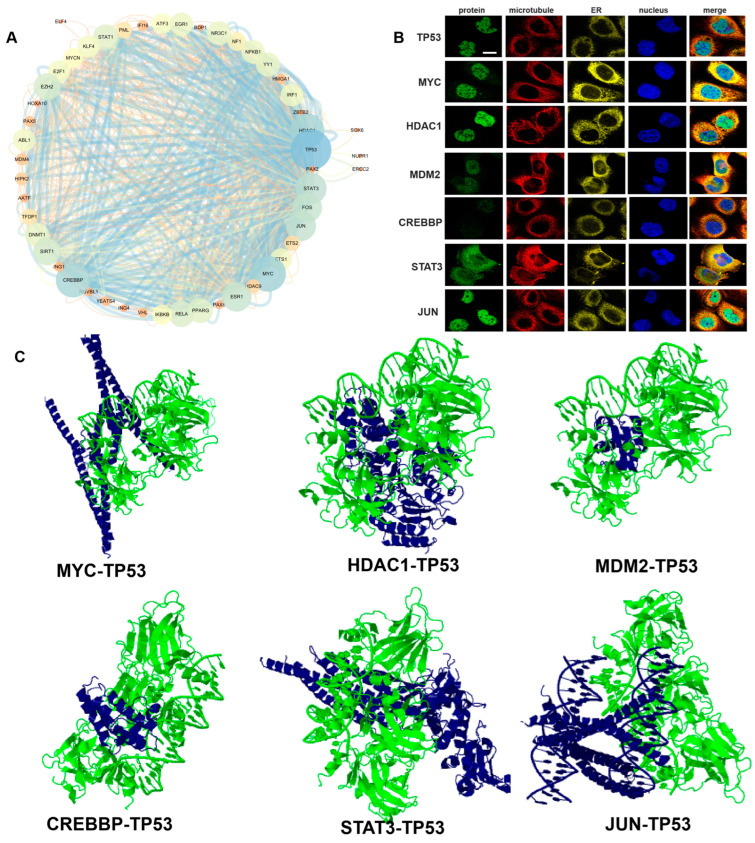
Analysis of *TP53*-related transcription factors. (**A**) PPI analysis of *TP53*-related transcription factors. (**B**) Location of *TP53*, MYC, HDAC1, MDM2, CREBBP, STAT3, and JUN proteins in cells: green represents the target protein, red represents microtubules, yellow represents the endoplasmic reticulum, and blue represents the nucleus (Scale bar, 10 μm). (**C**) *TP53* (1TUP) protein–protein docking results with MYC (6G6K), HDAC1 (4BKX), MDM2 (1YCR), CREBBP (5NLK), STAT3 (6NUQ), JUN (1FOS). Green represents the structure of *TP53* protein. Blue represents the protein structure of each transcription factor.

The *TP53* tumor suppressor protein is a major barrier preventing cancer from occurring and developing. In terms of biochemistry, *TP53* functions primarily as a sequence-specific transcription factor capable of binding to DNA sequences identified within the genome (called *TP53* response elements or *TP53* binding sites) and activating the transcription of adjacent genes, as well as the transcription of more distant genes regulated by enhancers with *TP53* binding sites. In addition, *TP53* can inhibit the transcription of a large number of genes, often through indirect mechanisms. In normal unstressed cells, *TP53* protein levels are kept low by conformational proteomic degradation, which is indicated by the E3 ubiquitin ligase MDM2, a major inhibitor of *TP53*. In addition, the biochemical activity of *TP53* as a transcription factor is also limited by MDM4 protein (also known as MDMX), meaning it is an additional physiological inhibitor of *TP53* [44]. We performed PPI analysis on *TP53*-related transcription factors, finding 51 nodes, 392 edges, a network density of 0.307, a network heterogeneity of 0.717, and a clustering coefficient of 0.685 (Figure 11A). As shown in Figure 11B, the protein expression of transcription factors *TP53*, MYC proto-oncogene, BHLH transcription factor (MYC), histone deacetylase 1 (HDAC1), MDM2, CREBBP, signal transducer and activator of transcription 3 (STAT3), and Jun proto-oncogene, AP-1 transcription factor subunit (JUN) were all located in the nucleoplasm. These proteins were subjected to molecular docking analysis with *TP53*, revealing that: MYC (6G6K) docked to *TP53* (1TUP), and the interacting amino acids included HIS, GLU, ASN, VAL, LYS, ARG, THR, GLN, LEU, SER, PHE, and ILE; HDAC1 (4BKX) docked to *TP53* (1TUP), and the amino acid sites were GLY, ILE, ARG, VAL, ASN, ARG, TYR, ALA, ASP, THR, LEU, LYS, GLU, and TRP; MDM2 (1YCR) docked to *TP53* (1TUP), and the amino acid sites were GLU, THR, LEU, VAL, ARG, PRO, LYS, GLN, ASP, TYR, MET, and PHE; CREBBP (5NLK) docked to *TP53* (1TUP), and the amino acid sites were LYS, ILE, PHE, PRO, GLU, ARG, GLN, ALA, MET, LEU, THR, TYR, and TRP; STAT3 (6NUQ) docked to *TP53* (1TUP), and the amino acid sites were VAL, THR, GLU, LYS, GLN, MET, LEU, HIS, ARG, GLN, ASP, SER, PHE, and ASN; and JUN (1FOS) docked to *TP53* (1TUP), and the amino acid sites were LYS, ARG, ILF, GLU, ASN, MET, ALA, SER, LEU, THR, ASP, GLN, and PHE (Figure 11C). The protein–protein docking interaction amino acid sites are shown in Supplementary Table S8.

### 3.13. Therapeutic Drugs and Molecular Docking

**Figure 12:**
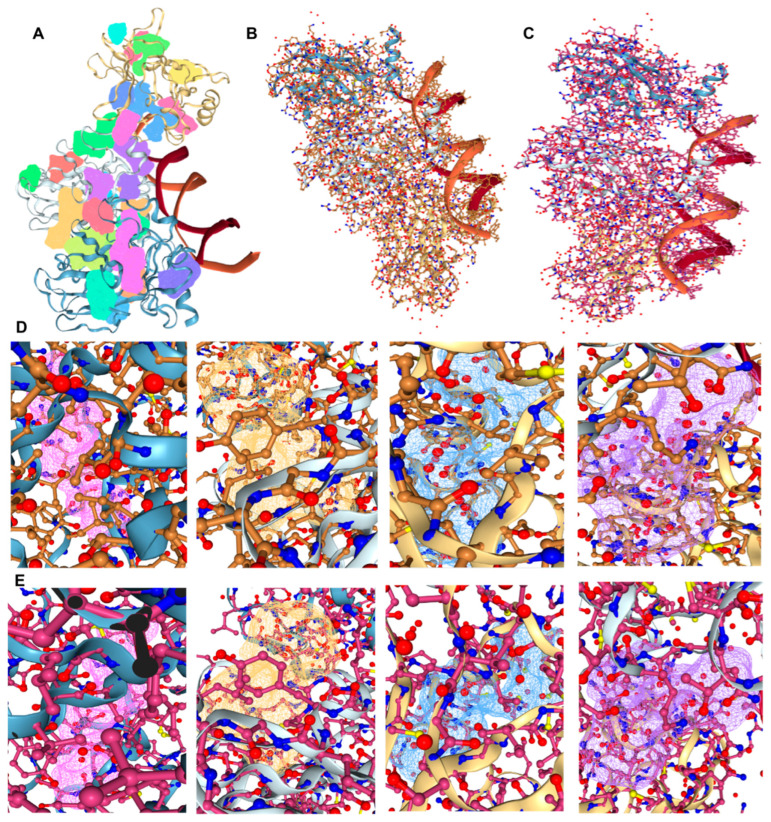
Molecular docking analysis of *TP53* and drugs. (**A**) *TP53* protein (1TUP) had 26 binding pockets. (**B**) Molecular docking results between *TP53* protein and pifithrin-mu. (**C**) Molecular docking results between *TP53* protein and aspirin. (**D**) The higher *TP53* protein drug score combined with the pocket (P_6, P_0, P_4, P_1) and pifithrin-mu molecular docking results. (**E**) The higher *TP53* protein drug score combined with the pocket (P_6, P_0, P_4, P_1) and aspirin molecular docking results.

The drugs that target *TP53* include pifithrin-mu, mepacrine, pifithrin-alpha, prima-1-met, and aspirin [45,46,47,48,49]. We predicted that *TP53* protein (1TUP) would have 26 pockets (Figure 12A, Supplementary Table S9), and we developed protein-binding pockets for molecular docking analysis with pifithrin-mu and aspirin (Figure 12B,C). Among these, the best binding sites for pifithrin-mu and aspirin to *TP53* protein were P_6, P_0, P_4, and P_1 (Figure 12D,E). The binding site scores and acting amino acids are listed in Table 3. It can be seen that the acting amino acids mainly included ALA, ARG, LEU, MET, SER, and VAL. Pifithrin-μ, a cell-permeable inhibitor of *TP53* binding and *TP53*-mediated apoptosis, directly inhibits *TP53* binding to the mitochondria [50] and inhibits *TP53* binding to Bcl-2 and Bcl-xL proteins [51]. Pifithrin-μ also selectively inhibits HSP70 activity. Pretreatment of mice with pifithrin-μ can repair the damage induced by γ-rays or DNA-damaging agents in primary thymic cells [51]. Aspirin regulates global demethylation and *TP53* activity and expression along with decreasing cell proliferation and migration [52]. Unfortunately, currently, there are no food and drug administration (FDA)-approved small-molecule drugs that directly target *TP53*. Currently, many targeted drugs and new treatment methods for *TP53* mutations are in clinical trials.

## 4. Discussion

The higher expression of *TP53* in tumors is an important indicator. TNM staging for tumors can determine the severity of disease and predict the survival rate of patients. High expression of *TP53* was associated with poor prognosis and tumor diagnosis, thus giving important information about patient disease progression and treatment response. In addition, mutations and methylation in *TP53* play an important role in the occurrence and development of tumors. The correlations between *TP53* expression and immune infiltration and immune checkpoints further indicated the potential role of *TP53* in tumor immunotherapy. *TP53* may play a crucial role in the biological processes of cancer progression, including showing positive correlations with differentiation, metastasis, inflammation, proliferation, and quiescence, and negative correlations with DNA repair, DNA damage, cell cycle, and apoptosis. Many transcription factors were associated with *TP53*, including AURKA, BARD1, CDK2, CREBBP, DDX5, EP300, GSK3B, KAT5, MDM2, and RPA1. These transcription factors, together with *TP53*, are involved in biological processes such as cell cycle regulation, DNA repair, apoptosis, cell migration, and metastasis, and they play an important regulatory role in the occurrence and development of tumors. For example, AURKA inhibits the DNA damage response by suppressing the expression of various DNA damage repair genes in a *TP53*-dependent manner [53]. MDM2 is a well-known transcription factor that interacts with *TP53* and can regulate the cell life cycle and apoptosis by binding to *TP53* and inhibiting its function [54]. In conclusion, there are complex interactions between *TP53* and these transcription factors, which play important regulatory roles in tumorigenesis. Studying the interaction mechanisms between these transcription factors and *TP53* is of great significance for understanding the tumorigenesis mechanism and providing targets and strategies for tumor therapy. In-depth research and comprehensive analyses of multiple aspects related to *TP53* may help to further elucidate the mechanisms underlying tumor occurrence and development and provide an important basis for precision treatment.

In many cancer processes, transcription factors can be mutated or dysregulated through various mechanisms of action, including chromosomal translocation, gene amplification or deletion, point mutations, and expression changes. The most prominent feature of *TP53* is that it is a transcription factor, and many of its target genes are related to apoptosis or cell cycle regulation, such as TP21-encoding cyclin-dependent protein kinase inhibitor and BAX-encoding apoptosis precursor protein [55]. Transcription factors play important biological roles in diseases such as cancer, autoimmune diseases, diabetes, and cardiovascular diseases [56]. However, transcription factors have traditionally been considered “untreatable” targets because of their severe structural disorganization and lack of well-defined small-molecule binding cavities [57]. Extensive experimental data have shown that mutated *TP53* plays a key role in promoting the malignant phenotype of cancers. Therefore, it is considered an attractive target for the treatment of various cancers. *TP53* gene testing aims to provide a more comprehensive assessment of the cancer risk of the subject [58], so that the patient can make timely and moderate adjustments to their lifestyle and living habits, such as exercise, diet, and sleep. *TP53* gene detection is a “cancer prevention guide” that can be used to help the subject actively avoid environmental risk factors that induce tumors, making the prevention and treatment of cancer more targeted and organized [59]. Although it is well established that *TP53* mutations affect cancer prognosis, they are rarely used for patient stratification or to guide treatment [60]. One of the important reasons for this is that the locations and types of *TP53* mutations have different effects on prognosis, and there is still a lack of unified classification criteria for *TP53* mutations. One classification classifies mutations into damaging and non-damaging mutations based on the degree of disruption of the *TP53* protein structure and function. Damaging mutations may result in complete or nearly complete loss of *TP53* protein activity. In contrast, non-destructive mutations can preserve some functional properties of the *TP53* protein.

Accumulating evidence suggests that *TP53* regulates both innate and acquired immune responses. *TP53* is an essential component of toll-like receptor 8 (TLR 8)-mediated immune responses. *TP53* is also involved in the activation of the major histocompatibility complex I (MHC-I) antigen presentation pathway by inducing transporter antigen peptide 1 (TAP1) [61,62]. However, mutations in *TP53* affect the recruitment and activity of T cells, leading to immune evasion and the promotion of cancer progression. In LUAD, mutant *TP53* inhibits the formation of the stimulator of interferon genes–TANK binding kinase 1–interferon regulatory factor 3 (STING-TBK1-IRF3) complex, leading to the inactivation of the innate immune signaling pathway [63]. Alternatively, mutant *TP53* has been found to be immunogenic, and could act as a novel antigen to trigger immune responses. For example, in LUAD, mutant *TP53* promotes PD-L1 expression and CD8+ T cell infiltration and enhances tumor immunogenicity [64]. Therefore, patients with mutant *TP53* may be more sensitive to PD-1 blockade immunotherapy. Studies have shown that patients with HNSC and destructive *TP53* mutations have a significantly shorter survival time, whereas patients with LUAD and non-destructive *TP53* mutations have worse prognosis [65]. Overall, although *TP53* mutations may make tumor treatment more difficult, investigators are actively searching for new treatments for *TP53*-mutated tumors. These novel therapies will provide more treatment options for patients with cancer and are expected to improve the efficacy of cancer treatment. It is believed that with the continuous progress of science and technology, we will derive a more effective means to fight cancer and bring hope to patients.

At present, the US FDA has approved some drugs targeting *TP53*. Gene therapy, targeted tumor vaccines, and anti-cancer drugs targeting *TP53* mutations are in the early stages of clinical trials, including APR-246 (eprenetapopt, PRIMA-1MET), PEITC (phenethyl isothiocyanate), ATO (arsenic trioxide/Trisenox), HSP90 inhibitor (ganetespib/STA-9090), Atorvastatin, Vorinostat/Zolinza/SAHA, Wee1 inhibitor (adavosertib/AZD1775/MK-1775), Lamivudine (3TC/Epivir/Zeffix/DELSTRIGO), Zoledronic acid (ZA/Reclast/Zometa), and atorvastatin [66,67]. Cancer cells with *TP53* mutations have also been shown to be sensitive to the Aurora A kinase inhibitor alisertib, which is a currently in a phase II clinical study for the treatment of non-small-cell lung carcinoma (NSCLC) [68]. In addition, studies have shown that *TP53* mutations may affect the efficacy of radiotherapy. Introducing wild-type *TP53* into tumor cells and reestablishing *TP53* protein function can help to increase tumor sensitivity to traditional radiotherapy and chemotherapy. This is understandable. Traditional treatment destroys DNA, and cancer cells with DNA damage are blocked by *TP53* protein and enter the apoptotic program. The cancer cells may have thus lost a checkpoint, or it could be that many tumors have *TP53* mutations, in part because of evolutionary selection.

However, several questions remain unanswered. First, *TP53* is mutated in more than 50% of tumors; therefore, what factors influence the type and spectrum of *TP53* mutations? Second, post-translational modifications play an important role in the accumulation of mutant *TP53*. How do post-translational modifications regulate the function of mutant *TP53* and what is the specific regulatory mechanism? Third, current studies have focused on mutational hotspots of *TP53*. It is uncertain whether *TP53* mutations with different residues and different functional domains exert the same gain of function, and what is the mechanism by which it exerts gain of function? Fourth, mutant *TP53* is generally considered to be “undruggable.” In recent years, although some studies have reported the development of a variety of small-molecule compounds or peptide drugs targeting mutant *TP53*, few drugs have entered clinical trials, and no drugs targeting mutant *TP53* have been approved for tumor treatment. Further research is needed on mutant *TP53*. There are also some limitations in this study. Bioinformatic analysis is an effective way to infer the role of a gene in a tumor, but it is not sufficient to fully confirm its role. Experimental verification can provide more reliable and concrete evidence to support the conclusions of bioinformatics analysis. For example, the expression of *TP53* in tumor cells can be disrupted or enhanced by CRISPR/Cas9 gene editing technology, after which the characteristics of cell proliferation, apoptosis, and invasion can be observed to further confirm the role of *TP53* in tumors.

## Figures and Tables

**Table 1:** Pan-cancer clinical significance analysis of *TP53*.

Tumor	Characteristics	Low Expression of *TP53*	High Expression of *TP53*	*p*-Value	Tumor	Characteristics	Low Expression of *TP53*	High Expression of *TP53*	*p*-Value	Tumor	Characteristics	Low Expression of *TP53*	High Expression of *TP53*	*p*-Value
ACC	n	39	40		BLCA	n	206	206		BRCA	n	543	544	
	Pathologic T stage, n (%)			0.135197547		Pathologic T stage, n (%)			0.596		Pathologic T stage, n (%)			0.741
	T1	7 (9.1%)	2 (2.6%)			T1	3 (0.8%)	2 (0.5%)			T1	138 (12.7%)	140 (12.9%)	
	T2	21 (27.3%)	21 (27.3%)			T2	54 (14.3%)	64 (16.9%)			T2	322 (29.7%)	309 (28.5%)	
	T3	2 (2.6%)	6 (7.8%)			T3	103 (27.2%)	93 (24.6%)			T3	65 (6%)	75 (6.9%)	
	T4	7 (9.1%)	11 (14.3%)			T4	32 (8.5%)	27 (7.1%)			T4	16 (1.5%)	19 (1.8%)	
	Pathologic N stage, n (%)			1		Pathologic N stage, n (%)			0.212		Pathologic N stage, n (%)			0.627
	N0	33 (42.9%)	35 (45.5%)			N0	110 (29.9%)	128 (34.8%)			N0	268 (25.1%)	248 (23.2%)	
	N1	4 (5.2%)	5 (6.5%)			N1	24 (6.5%)	22 (6%)			N1	174 (16.3%)	185 (17.3%)	
	Clinical M stage, n (%)			0.486682972		N2	46 (12.5%)	31 (8.4%)			N2	55 (5.1%)	61 (5.7%)	
	M0	31 (40.3%)	31 (40.3%)			N3	3 (0.8%)	4 (1.1%)			N3	36 (3.4%)	41 (3.8%)	
	M1	6 (7.8%)	9 (11.7%)			Pathologic M stage, n (%)			0.138		Pathologic M stage, n (%)			0.395
CESC	n	153	153			M0	92 (43.4%)	109 (51.4%)			M0	449 (48.5%)	456 (49.3%)	
	Pathologic T stage, n (%)			0.996		M1	2 (0.9%)	9 (4.2%)			M1	8 (0.9%)	12 (1.3%)	
	T1	66 (27.2%)	74 (30.5%)		CHOL	n	17	18		COAD	n	239	239	
	T2	35 (14.4%)	37 (15.2%)			Pathologic T stage, n (%)			0.103		Pathologic T stage, n (%)			0.603
	T3	10 (4.1%)	11 (4.5%)			T1	12 (34.3%)	6 (17.1%)			T1	6 (1.3%)	5 (1%)	
	T4	5 (2.1%)	5 (2.1%)			T2	4 (11.4%)	8 (22.9%)			T3	168 (35.2%)	155 (32.5%)	
	Pathologic N stage, n (%)			0.567		T3	1 (2.9%)	4 (11.4%)			T4	28 (5.9%)	32 (6.7%)	
	N0	60 (30.8%)	74 (37.9%)			T4	0 (0%)	0 (0%)			T2	37 (7.8%)	46 (9.6%)	
	N1	30 (15.4%)	31 (15.9%)			Pathologic N stage, n (%)			0.33		Pathologic N stage, n (%)			0.017
	Pathologic M stage, n (%)			0.915		N0	14 (46.7%)	11 (36.7%)			N0	134 (28%)	150 (31.4%)	
	M0	57 (22.3%)	59 (23%)			N1	1 (3.3%)	4 (13.3%)			N1	50 (10.5%)	58 (12.1%)	
	M1	6 (2.3%)	5 (2%)			Pathologic M stage, n (%)			1		N2	55 (11.5%)	31 (6.5%)	
	MX	62 (24.2%)	67 (26.2%)			M0	13 (40.6%)	14 (43.8%)			Pathologic M stage, n (%)			0.905
DLBC	n	24	24			M1	2 (6.2%)	3 (9.4%)			M0	177 (42.7%)	172 (41.4%)	
	Clinical stage, n (%)			0.081	ESCA	n	81	82			M1	34 (8.2%)	32 (7.7%)	
	Stage I	4 (9.5%)	4 (9.5%)			Pathologic T stage, n (%)			0.518	HNSC	n	252	252	
	Stage II	6 (14.3%)	11 (26.2%)			T1	16 (11%)	11 (7.6%)			Pathologic T stage, n (%)			0.488
	Stage III	5 (11.9%)	0 (0%)			T2	19 (13.1%)	18 (12.4%)			T1	23 (5.1%)	22 (4.9%)	
	Stage IV	5 (11.9%)	7 (16.7%)			T3	36 (24.8%)	41 (28.3%)			T2	64 (14.3%)	71 (15.8%)	
KICH	n	32	33			T4	1 (0.7%)	3 (2.1%)			T3	47 (10.5%)	49 (10.9%)	
	Pathologic T stage, n (%)			0.338		Pathologic N stage, n (%)			0.398		T4	96 (21.4%)	76 (17%)	
	T1	8 (12.3%)	12 (18.5%)			N0	28 (19.4%)	38 (26.4%)			Pathologic N stage, n (%)			0.731
	T2	14 (21.5%)	11 (16.9%)			N1	35 (24.3%)	28 (19.4%)			N0	94 (22.9%)	77 (18.7%)	
	T3	10 (15.4%)	8 (12.3%)			N2	5 (3.5%)	4 (2.8%)			N1	33 (8%)	33 (8%)	
	T4	0 (0%)	2 (3.1%)			N3	2 (1.4%)	4 (2.8%)			N2	83 (20.2%)	84 (20.4%)	
	Pathologic N stage, n (%)			0.675		Pathologic M stage, n (%)			1		N3	3 (0.7%)	4 (1%)	
	N0	23 (52.3%)	16 (36.4%)			M0	60 (46.5%)	61 (47.3%)			Pathologic M stage, n (%)			0.45
	N1	1 (2.3%)	2 (4.5%)			M1	4 (3.1%)	4 (3.1%)			M0	104 (55%)	84 (44.4%)	
	N2	1 (2.3%)	1 (2.3%)		KIRC	n	270	271			M1	0 (0%)	1 (0.5%)	
	Pathologic M stage, n (%)			1		Pathologic T stage, n (%)			0.017	KIRP	n	145	146	
	M0	19 (52.8%)	15 (41.7%)			T1	128 (23.7%)	151 (27.9%)			Pathologic T stage, n (%)			0.232
	M1	1 (2.8%)	1 (2.8%)			T2	47 (8.7%)	24 (4.4%)			T1	102 (35.3%)	91 (31.5%)	
LIHC	n	187	187			T3	91 (16.8%)	89 (16.5%)			T2	17 (5.9%)	17 (5.9%)	
	Pathologic T stage, n (%)			0.254		T4	4 (0.7%)	7 (1.3%)			T3	25 (8.7%)	35 (12.1%)	
	T1	98 (26.4%)	85 (22.9%)			Pathologic N stage, n (%)			0.036		T4	0 (0%)	2 (0.7%)	
	T2	47 (12.7%)	48 (12.9%)			N0	126 (48.8%)	116 (45%)			Pathologic N stage, n (%)			0.011
	T3	33 (8.9%)	47 (12.7%)			N1	4 (1.6%)	12 (4.7%)			N0	29 (37.2%)	21 (26.9%)	
	T4	8 (2.2%)	5 (1.3%)			Pathologic M stage, n (%)			0.771		N1	5 (6.4%)	19 (24.4%)	
	Pathologic N stage, n (%)			0.659		M0	214 (42.1%)	215 (42.3%)			N2	2 (2.6%)	2 (2.6%)	
	N0	124 (48.1%)	130 (50.4%)			M1	38 (7.5%)	41 (8.1%)			Pathologic M stage, n (%)			1
	N1	1 (0.4%)	3 (1.2%)		LUAD	n	269	270			M0	48 (46.2%)	47 (45.2%)	
	Pathologic M stage, n (%)			0.625		Pathologic T stage, n (%)			0.554		M1	4 (3.8%)	5 (4.8%)	
	M0	134 (49.3%)	134 (49.3%)			T1	83 (15.5%)	93 (17.4%)		LUSC	n	251	251	
	M1	3 (1.1%)	1 (0.4%)			T2	148 (27.6%)	144 (26.9%)			Pathologic T stage, n (%)			0.41
MESO	n	43	44			T3	28 (5.2%)	21 (3.9%)			T1	60 (12%)	54 (10.8%)	
	Pathologic T stage, n (%)			0.464		T4	8 (1.5%)	11 (2.1%)			T2	142 (28.3%)	152 (30.3%)	
	T1	7 (8.2%)	7 (8.2%)			Pathologic N stage, n (%)			0.193		T3	40 (8%)	31 (6.2%)	
	T2	12 (14.1%)	14 (16.5%)			N0	184 (35.2%)	166 (31.7%)			T4	9 (1.8%)	14 (2.8%)	
	T3	14 (16.5%)	18 (21.2%)			N1	43 (8.2%)	54 (10.3%)			Pathologic N stage, n (%)			0.635
	T4	9 (10.6%)	4 (4.7%)			N2	30 (5.7%)	44 (8.4%)			N0	160 (32.3%)	160 (32.3%)	
	Pathologic N stage, n (%)			0.063		N3	1 (0.2%)	1 (0.2%)			N1	61 (12.3%)	70 (14.1%)	
	N0	18 (21.7%)	26 (31.3%)			Pathologic M stage, n (%)			0.571		N2	23 (4.6%)	17 (3.4%)	
	N1	4 (4.8%)	6 (7.2%)			M0	182 (46.7%)	183 (46.9%)			N3	3 (0.6%)	2 (0.4%)	
	N2	17 (20.5%)	9 (10.8%)			M1	11 (2.8%)	14 (3.6%)			Pathologic M stage, n (%)			0.473
	N3	3 (3.6%)	0 (0%)		PAAD	n	89	90			M0	208 (49.6%)	204 (48.7%)	
	Pathologic M stage, n (%)			1		Pathologic T stage, n (%)			0.515		M1	5 (1.2%)	2 (0.5%)	
	M0	28 (46.7%)	29 (48.3%)			T1	5 (2.8%)	2 (1.1%)		PRAD	n	250	251	
	M1	2 (3.3%)	1 (1.7%)			T3	71 (40.1%)	72 (40.7%)			Pathologic T stage, n (%)			0.186
READ	n	83	83			T4	2 (1.1%)	1 (0.6%)			T2	87 (17.6%)	102 (20.6%)	
	Pathologic T stage, n (%)			0.162		T2	10 (5.6%)	14 (7.9%)			T3	157 (31.8%)	137 (27.7%)	
	T1	2 (1.2%)	7 (4.3%)			Pathologic N stage, n (%)			0.887		T4	4 (0.8%)	7 (1.4%)	
	T3	62 (37.8%)	51 (31.1%)			N0	24 (13.8%)	26 (14.9%)			Pathologic N stage, n (%)			0.804
	T4	7 (4.3%)	7 (4.3%)			N1	61 (35.1%)	63 (36.2%)			N0	173 (40.4%)	175 (40.9%)	
	T2	11 (6.7%)	17 (10.4%)			Pathologic M stage, n (%)			1		N1	41 (9.6%)	39 (9.1%)	
	Pathologic N stage, n (%)			0.963		M0	34 (40%)	46 (54.1%)		STAD	n	187	188	
	N0	43 (26.5%)	41 (25.3%)			M1	2 (2.4%)	3 (3.5%)			Pathologic T stage, n (%)			0.416
	N1	22 (13.6%)	23 (14.2%)		SKCM	n	236	236			T1	11 (3%)	8 (2.2%)	
	N2	17 (10.5%)	16 (9.9%)			Pathologic T stage, n (%)			0.848		T3	87 (23.7%)	81 (22.1%)	
	Pathologic M stage, n (%)			0.794		T1	23 (6.3%)	19 (5.2%)			T4	43 (11.7%)	57 (15.5%)	
	M0	62 (41.6%)	64 (43%)			T2	39 (10.7%)	40 (11%)			T2	42 (11.4%)	38 (10.4%)	
	M1	12 (8.1%)	11 (7.4%)			T3	43 (11.8%)	48 (13.2%)			Pathologic N stage, n (%)			0.303
TGCT	n	69	70			T4	79 (21.6%)	74 (20.3%)			N0	59 (16.5%)	52 (14.6%)	
	Pathologic T stage, n (%)			0.682		Pathologic N stage, n (%)			0.24		N1	41 (11.5%)	56 (15.7%)	
	T1	39 (28.3%)	41 (29.7%)			N0	108 (26%)	128 (30.8%)			N2	37 (10.4%)	38 (10.6%)	
	T2	27 (19.6%)	25 (18.1%)			N1	42 (10.1%)	32 (7.7%)			N3	41 (11.5%)	33 (9.2%)	
	T3	2 (1.4%)	4 (2.9%)			N2	25 (6%)	24 (5.8%)			Pathologic M stage, n (%)			0.056
	Pathologic N stage, n (%)			0.601		N3	32 (7.7%)	24 (5.8%)			M0	159 (44.8%)	171 (48.2%)	
	N0	16 (25%)	35 (54.7%)			Pathologic M stage, n (%)			0.568		M1	17 (4.8%)	8 (2.3%)	
	N1	5 (7.8%)	6 (9.4%)			M0	209 (47.1%)	210 (47.3%)		UVM	n	40	40	
	N2	1 (1.6%)	1 (1.6%)			M1	11 (2.5%)	14 (3.2%)			Pathologic M stage, n (%)			0.835
	Pathologic M stage, n (%)			0.153	THCA	n	256	256			M0	25 (32.1%)	26 (33.3%)	
	M0	59 (47.6%)	61 (49.2%)			Pathologic T stage, n (%)			0.043		M1	2 (2.6%)	2 (2.6%)	
	M1	0 (0%)	4 (3.2%)			T1	68 (13.3%)	75 (14.7%)						
						T2	73 (14.3%)	96 (18.8%)						
						T3	100 (19.6%)	75 (14.7%)						
						T4	14 (2.7%)	9 (1.8%)						
						Pathologic N stage, n (%)			0.077					
						N0	104 (22.5%)	125 (27.1%)						
						N1	125 (27.1%)	108 (23.4%)						
						Pathologic M stage, n (%)			0.214					
						M0	140 (47.5%)	146 (49.5%)						
						M1	2 (0.7%)	7 (2.4%)						

**Table 2:** Analysis of the logistics model between clinical pathological staging and *TP53*.

Tumor	Characteristics	Total (N)	OR (95% CI)	*p*-Value	Tumor	Characteristics	Total (N)	OR (95% CI)	*p*-Value	Tumor	Characteristics	Total (N)	OR (95% CI)	*p*-Value
ACC	Pathologic T stage (T3&T4 vs. T1&T2)	77	2.300 (0.865–6.116)	0.095	BLCA	Pathologic T stage (T3&T4 vs. T1&T2)	378	0.768 (0.499–1.182)	0.23	BRCA	Pathologic T stage (T2&T3&T4 vs. T1)	1084	0.986 (0.750–1.295)	0.918
	Pathologic N stage (N1 vs. N0)	77	1.179 (0.291–4.770)	0.818		Pathologic N stage (N1&N2&N3 vs. N0)	368	0.671 (0.436–1.032)	0.069		Pathologic N stage (N1&N2&N3 vs. N0)	1068	1.170 (0.920–1.488)	0.199
CESC	Pathologic T stage (T2&T3&T4 vs. T1)	243	0.945 (0.568–1.573)	0.829		Pathologic M stage (M1 vs. M0)	212	3.798 (0.800–18.022)	0.093		Pathologic M stage (M1 vs. M0)	925	1.477 (0.598–3.647)	0.398
	Pathologic N stage (N1 vs. N0)	195	0.838 (0.457–1.537)	0.568	CHOL	Pathologic T stage (T2&T3&T4 vs. T1)	35	4.800 (1.147–20.085)	0.032	COAD	Pathologic T stage (T3&T4 vs. T1&T2)	477	0.804 (0.512–1.265)	0.346
	Pathologic M stage (M1&MX vs. M0)	256	1.023 (0.625–1.674)	0.928		Pathologic N stage (N1 vs. N0)	30	5.091 (0.496–52.285)	0.171		Pathologic N stage (N1&N2 vs. N0)	478	0.757 (0.525–1.092)	0.136
ESCA	Pathologic T stage (T3&T4 vs. T1&T2)	145	1.435 (0.743–2.772)	0.282		Pathologic M stage (M1 vs. M0)	32	1.393 (0.200–9.711)	0.738		Pathologic M stage (M1 vs. M0)	415	0.969 (0.572–1.640)	0.905
	Pathologic N stage (N1&N2&N3 vs. N0)	144	0.632 (0.326–1.223)	0.173	HNSC	Pathologic T stage (T3&T4 vs. T1&T2)	448	0.818 (0.560–1.194)	0.297	KICH	Pathologic T stage (T2&T3&T4 vs. T1)	65	0.583 (0.200–1.699)	0.323
KIRC	Pathologic T stage (T2&T3&T4 vs. T1)	541	0.716 (0.511–1.005)	0.053		Pathologic N stage (N1&N2&N3 vs. N0)	411	1.241 (0.838–1.840)	0.282		Pathologic N stage (N1&N2 vs. N0)	44	2.156 (0.323–14.410)	0.428
	Pathologic N stage (N1 vs. N0)	258	3.259 (1.022–10.388)	0.046		Pathologic M stage (M1 vs. M0)	189	75169169.1684 (0.000–Inf)	0.997		Pathologic M stage (M1 vs. M0)	36	1.267 (0.073–21.968)	0.871
	Pathologic M stage (M1 vs. M0)	508	1.074 (0.664–1.736)	0.771	KIRP	Pathologic T stage (T2&T3&T4 vs. T1)	289	1.441 (0.881–2.358)	0.146	LIHC	Pathologic T stage (T2&T3&T4 vs. T1)	371	1.310 (0.871–1.970)	0.194
LUAD	Pathologic T stage (T2&T3&T4 vs. T1)	536	0.854 (0.595–1.225)	0.390		Pathologic N stage (N1&N2 vs. N0)	78	4.143 (1.489–11.527)	0.006		Pathologic N stage (N1 vs. N0)	258	2.862 (0.294–27.877)	0.365
	Pathologic N stage (N1&N2&N3 vs. N0)	523	1.483 (1.027–2.141)	0.035		Pathologic M stage (M1 vs. M0)	104	1.277 (0.323–5.049)	0.728		Pathologic M stage (M1 vs. M0)	272	0.333 (0.034–3.245)	0.344
	Pathologic M stage (M1 vs. M0)	390	1.266 (0.560–2.862)	0.571	LUSC	Pathologic T stage (T2&T3&T4 vs. T1)	502	1.146 (0.754–1.741)	0.523	MESO	Pathologic T stage (T3&T4 vs. T1&T2)	85	0.865 (0.369–2.030)	0.74
PAAD	Pathologic T stage (T3&T4 vs. T1&T2)	177	0.937 (0.432–2.036)	0.870		Pathologic N stage (N1&N2&N3 vs. N0)	496	1.023 (0.708–1.478)	0.904		Pathologic N stage (N1&N2&N3 vs. N0)	83	0.433 (0.179–1.045)	0.063
	Pathologic N stage (N1 vs. N0)	174	0.953 (0.494–1.839)	0.887		Pathologic M stage (M1 vs. M0)	419	0.408 (0.078–2.126)	0.287		Pathologic M stage (M1 vs. M0)	60	0.483 (0.041–5.628)	0.561
	Pathologic M stage (M1 vs. M0)	85	1.109 (0.176–7.004)	0.913	PRAD	Pathologic T stage (T3&T4 vs. T2)	494	0.763 (0.530–1.098)	0.145	READ	Pathologic T stage (T3&T4 vs. T1&T2)	164	0.455 (0.213–0.973)	0.042
SKCM	Pathologic T stage (T3&T4 vs. T1&T2)	365	1.051 (0.680–1.625)	0.824		Pathologic N stage (N1 vs. N0)	428	0.940 (0.578–1.529)	0.804		Pathologic N stage (N1&N2 vs. N0)	162	1.049 (0.566–1.943)	0.880
	Pathologic N stage (N1&N2&N3 vs. N0)	415	0.682 (0.461–1.007)	0.054	STAD	Pathologic T stage (T3&T4 vs. T1&T2)	367	1.223 (0.771–1.941)	0.393		Pathologic M stage (M1 vs. M0)	149	0.888 (0.365–2.162)	0.794
	Pathologic M stage (M1 vs. M0)	444	1.267 (0.562–2.855)	0.569		Pathologic N stage (N1&N2&N3 vs. N0)	357	1.211 (0.773–1.897)	0.403	UVM	Pathologic M stage (M1&MX vs. M0)	78	0.769 (0.301–1.963)	0.583
TGCT	Pathologic T stage (T2&T3 vs. T1)	138	0.951 (0.484–1.870)	0.885		Pathologic M stage (M1 vs. M0)	355	0.438 (0.184–1.042)	0.062					
	Pathologic N stage (N1&N2 vs. N0)	64	0.533 (0.154–1.844)	0.321	THCA	Pathologic T stage (T3&T4 vs. T1&T2)	510	0.608 (0.424–0.870)	0.007					
	Pathologic M stage (M1 vs. M0)	124	56035110.1225 (0.000–Inf)	0.994		Pathologic N stage (N1 vs. N0)	462	0.719 (0.499–1.037)	0.077					
						Pathologic M stage (M1 vs. M0)	295	3.356 (0.685–16.433)	0.135					

**Table 3:** The best binding pockets of *TP53* protein.

Name	P_6	P_0	P_4	P_1
drugScore	0.785028	0.745974	0.723506	0.680187
volume	277.86	485.15	309.3	419.38
surface	312.4	685.27	243.85	613
depth	21.1	16.15	18.27	15.25
surf/vol	1.124307205	1.412490982	0.788393146	1.46168153
ell c/a	0.05	0.09	0.1	0.11
ell b/a	0.14	0.2	0.29	0.79
siteAtms	135	97	145	124
accept	34	25	42	33
donor	16	12	15	11
hydrophobicity	0.18	0.53	0.08	0.37
Cs	92	67	97	81
Ns	25	12	26	23
Os	16	17	17	19
Ss	2	1	5	1
Xs	0	0	0	0
negAA	0.04	0.09	0.03	0.07
posAA	0.24	0.18	0.25	0.26
polarAA	0.2	0.27	0.38	0.37
apolarAA	0.52	0.45	0.34	0.3
ALA	3	1	2	2
ARG	4	3	5	3
ASN	0	0	2	1
ASP	1	1	0	1
CYS	0	0	4	2
GLN	1	0	1	2
GLU	0	1	1	1
GLY	0	0	2	0
HIS	2	0	3	3
ILE	2	3	2	0
LEU	1	2	1	1
LYS	0	1	0	1
MET	2	1	2	2
PHE	0	0	0	0
PRO	2	1	1	0
SER	3	2	1	4
THR	0	3	0	1
TRP	0	0	0	0
TYR	1	1	2	0
VAL	3	2	3	3

## Supplementary Materials

The following supporting information can be downloaded at: https://www.mdpi.com/article/10.3390/cimb45120601/s1, Figure S1 Aberrant expression of *TP53* in pan-cancer. (A) Box plot of the *TP53* mRNA level analyzed by the GEPIA2 database. (B) The expression of *TP53* in normal tissue and BRCA, GBM, and STAD performed by CPTAC. (C) Tumors with no clear association between *TP53* expression and patients’ stage. Figure S2 RNA-sequencing and TCGA data were used to analyze the survival and RFS. (A) The high SLC31A1 RNA-sequencing expression level was associated with poor prognosis in patients with BRCA and COAD. (B) TCGA data analysis OS and RFS of BRCA, COAD, LGG, PRAD. Figure S3 The mutation landscape of SLC31A1 in pan-cancer. Figure S4 Information of *TP53* mutation samples and cohorts from the cBioPortal tool. Figure S5 Analysis of immune infiltration, tumor dryness, and immune checkpoint. (A) Correlation heatmap between *TP53* expression and tumor infifiltrating immune cells across different cancer types was displayed, including Neutrophil, Mast cell, NK cell, and Macrophage. (B) *TP53* expression and tumor stemness, a significant positive correlation in 3 tumors (LGG, LAML, and THYM) and a significant negative correlation in 11 tumors ( BRCA, SARC, KIRP, KIPAN, KIRC, LIHC, THCA, TGCT, PCPG, SKCM, KICH ). (C) Correlation between *TP53* and 60 immune checkpoint (inhibitory, stimulatory). * *p* < 0.05. Figure S6 (A) *TP53*-correlated genes analyzed by GEPIA2, including AURKA, BARD1, CDK2, CREBBP, DDX5, EP300, GSK3B, KAT5, MDM2, and RPA1. (B) Survival curve analysis of hsa-let-7e-5p, hsa-miR-98-5p, hsa-miR-221-3p, hsa-miR-222-3p, hsa-miR-380-5p, and hsa-miR-485-5p in pan-cancer. Figure S7 *TP53*-associated signaling pathways in pan-cancer.
