# Supplementary material for: Integrative Analysis of the Role of TP53 in Human Pan-Cancer
Source: Curr Issues Mol Biol. 2023 Nov 29;45(12):9606–33. doi: 10.3390/cimb45120601 (PMC10742156; doi:10.3390/cimb45120601)
Supplement: Supplementary file 1 [file cimb-45-00601-s001.zip › cimb-2706635-supplementary.pdf]

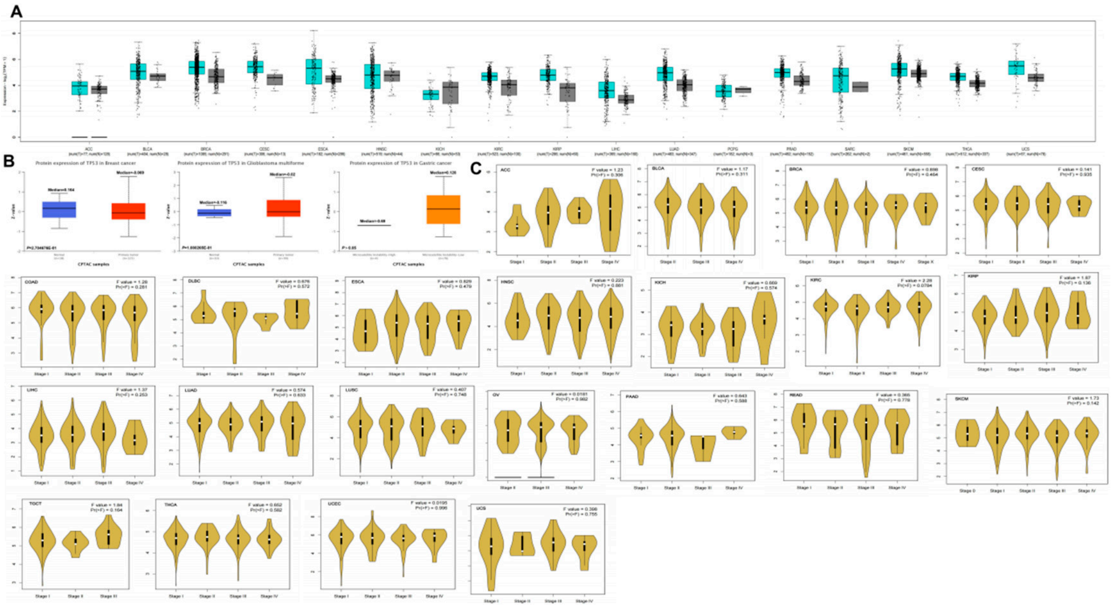

Figure S1 Aberrant expression of TP53 in pan-cancer. (A) Box plot of the TP53 mRNA level analyzed by the GEPIA2 database. (B) The expression of TP53 in normal tissue and BRCA, GBM, and STAD performed by CPTAC. (C) Tumors with no clear association between TP53 expression and patients' stage.

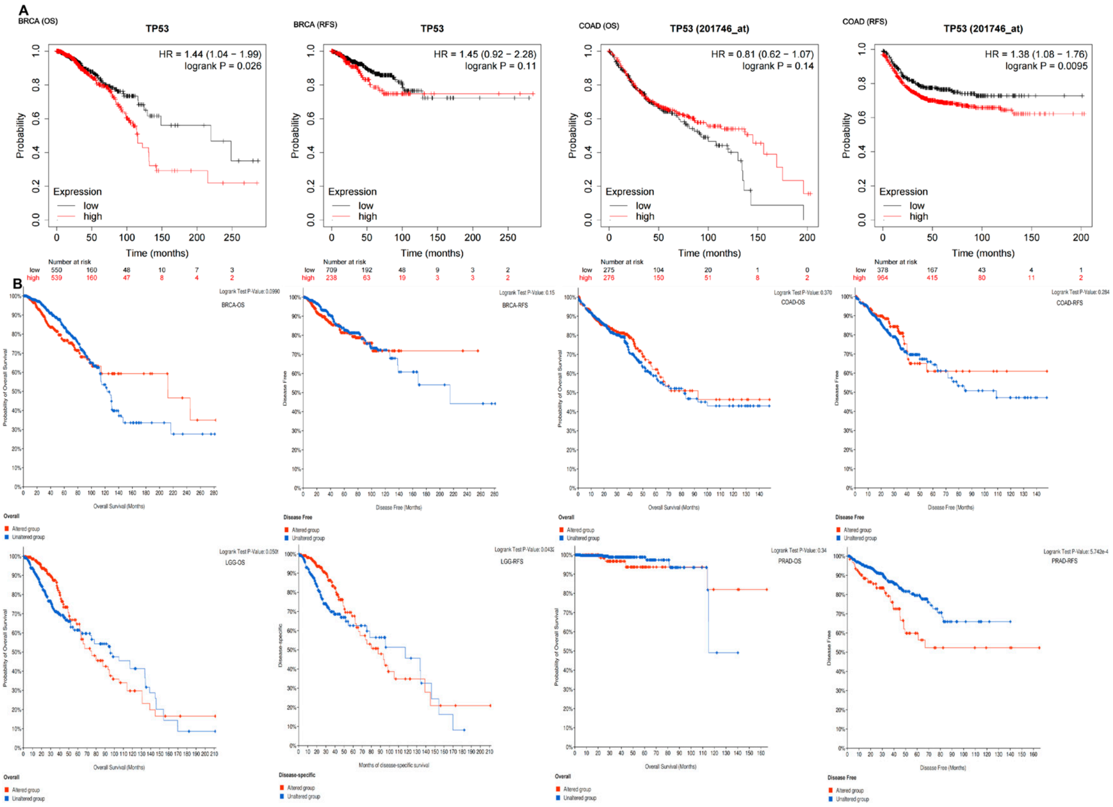

Figure S2 RNA-sequencing and TCGA data were used to analyze the survival and RFS. (A) The high SLC31A1 RNA-sequencing expression level was associated with poor prognosis in patients with BRCA and COAD. (B) TCGA data analysis OS and RFS of BRCA, COAD, LGG, PRAD.

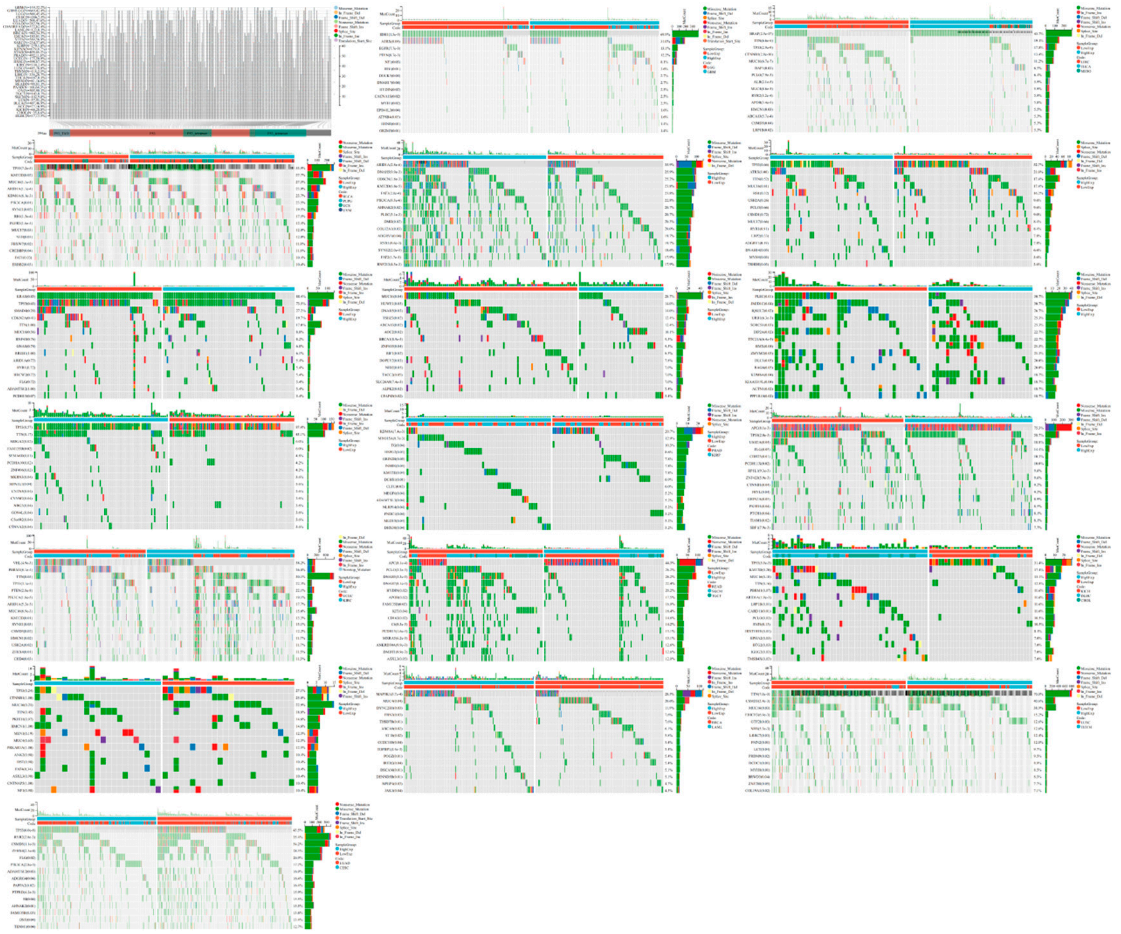

Figure S3 The mutation landscape of SLC31A1 in pan-cancer.

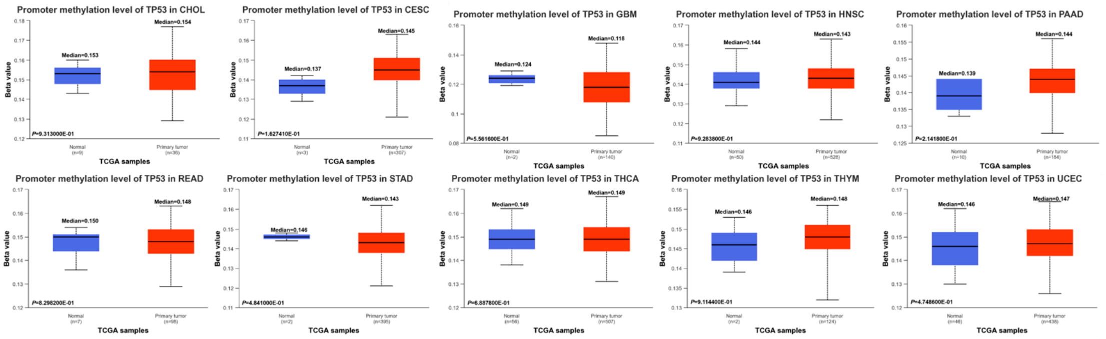

Figure S4 Information of TP53 mutation samples and cohorts from the cBioPortal tool.

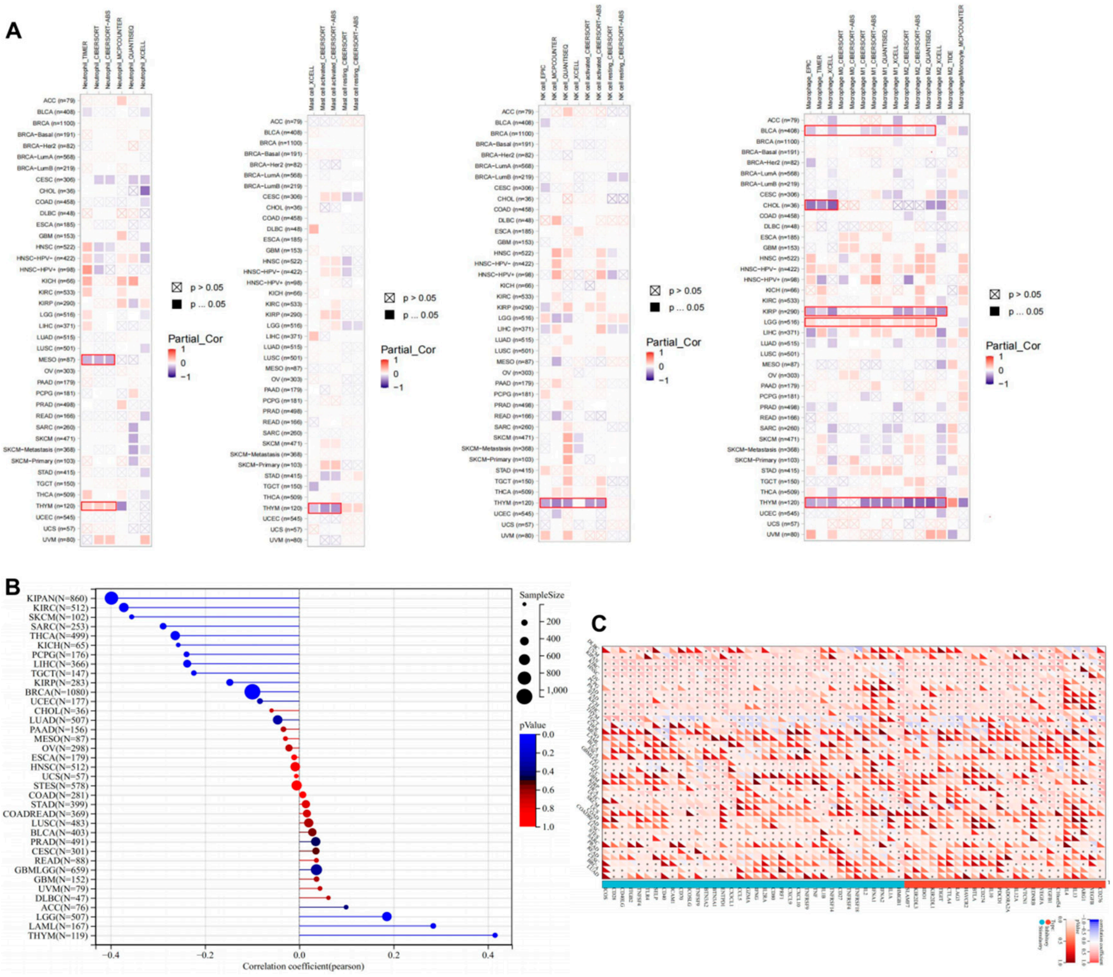

Figure S5 Analysis of immune infiltration, tumor dryness, and immune checkpoint. (A) Correlation heatmap between TP53 expression and tumor infifiltrating immune cells across different cancer types was displayed, including Neutrophil, Mast cell, NK cell, and Macrophage. (B) TP53 expression and tumor stemness, a significant positive correlation in 3 tumors (LGG, LAML, and THYM) and a significant negative correlation in 11 tumors (BRCA, SARC, KIRP, KIPAN, KIRC, LIHC, THCA, TGCT, PCPG, SKCM, KICH). (C) Correlation between TP53 and 60 immune checkpoint (inhibitory, stimulatory). *$p < 0.05$.

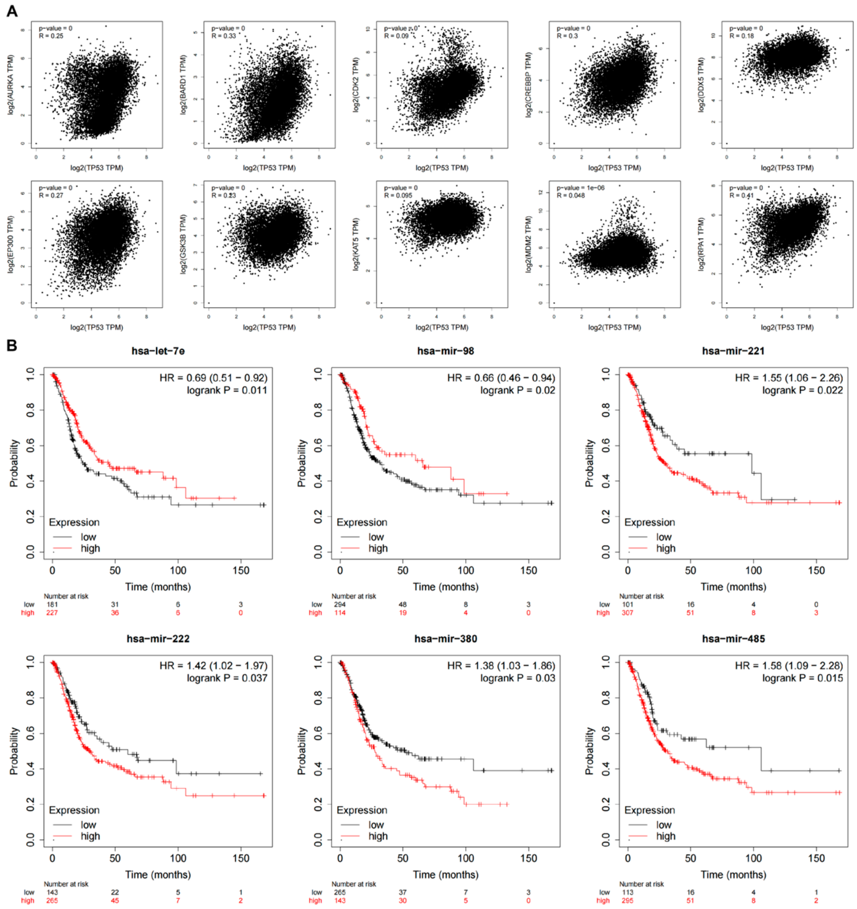

Figure S6 (A) TP53-correlated genes analyzed by GEPIA2, including AURKA, BARD1, CDK2, CREBBP, DDX5, EP300, GSK3B, KAT5, MDM2, and RPA1. (B) Survival curve analysis of hsa-let-7e-5p, hsa-miR-98-5p, hsa-miR-221-3p, hsa-miR-222-3p, hsa-miR-380-5p, and hsa-miR-485-5p in pan-cancer.

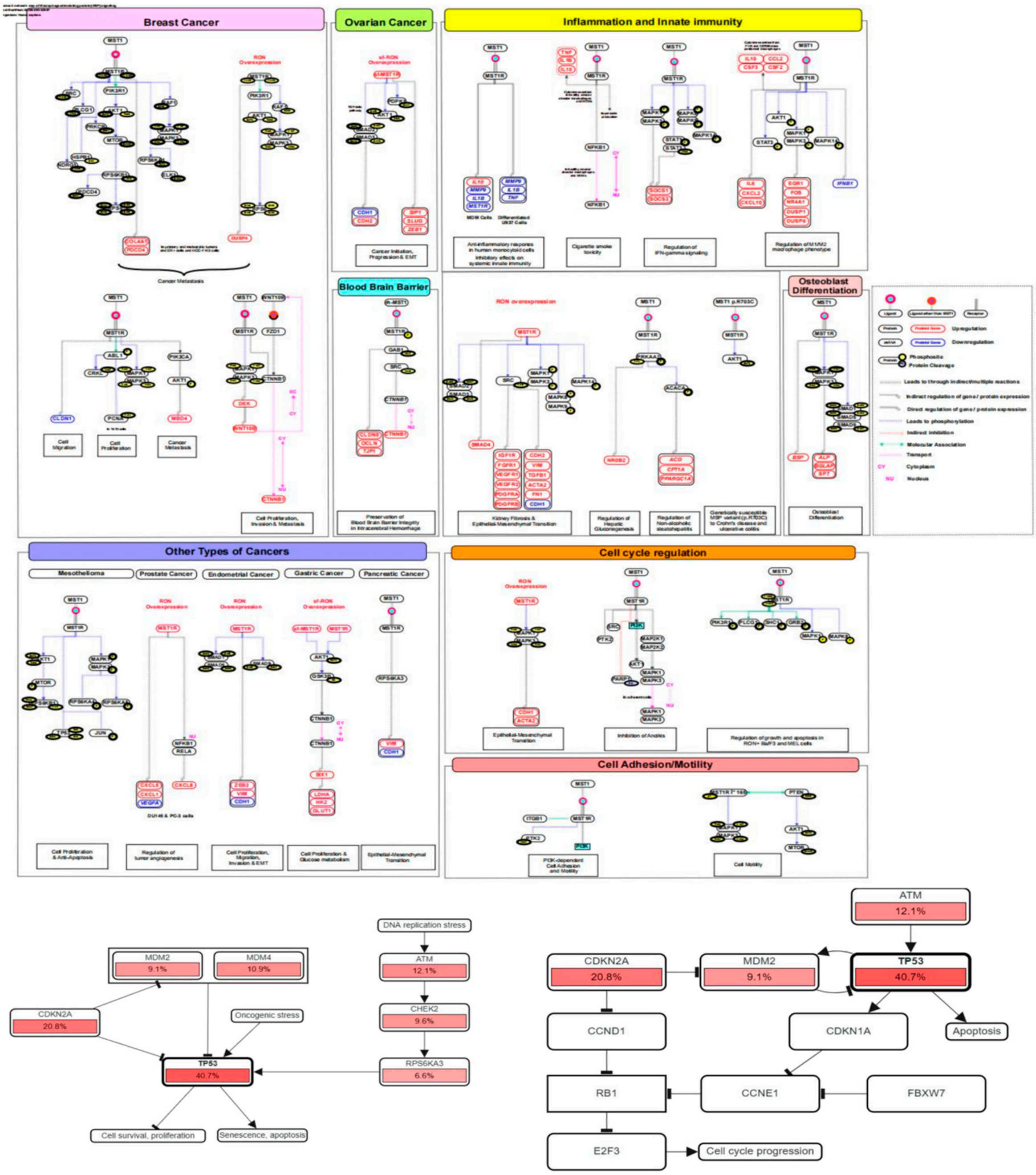

Figure S7 TP53-associated signaling pathways in pan-cancer.
